# Genetic Basis for Variation in Wheat Grain Yield in Response to Varying Nitrogen Application

**DOI:** 10.1371/journal.pone.0159374

**Published:** 2016-07-26

**Authors:** Saba Mahjourimajd, Julian Taylor, Beata Sznajder, Andy Timmins, Fahimeh Shahinnia, Zed Rengel, Hossein Khabaz-Saberi, Haydn Kuchel, Mamoru Okamoto, Peter Langridge

**Affiliations:** 1 Australian Centre for Plant Functional Genomics (ACPFG), The University of Adelaide, PMB1, Glen Osmond, SA, 5064, Australia; 2 Australian Grain Technologies, PMB1, Glen Osmond, SA, 5064, Australia; 3 School of Agriculture, Food and Wine, Waite Research Institute, The University of Adelaide, PMB 1, Glen Osmond, SA, 5064, Australia; 4 Soil Science and Plant Nutrition M087, School of Earth and Environment, University of Western Australia, 35 Stirling Highway, Crawley, WA, 6009, Australia; University of Missouri, UNITED STATES

## Abstract

Nitrogen (N) is a major nutrient needed to attain optimal grain yield (GY) in all environments. Nitrogen fertilisers represent a significant production cost, in both monetary and environmental terms. Developing genotypes capable of taking up N early during development while limiting biomass production after establishment and showing high N-use efficiency (NUE) would be economically beneficial. Genetic variation in NUE has been shown previously. Here we describe the genetic characterisation of NUE and identify genetic loci underlying N response under different N fertiliser regimes in a bread wheat population of doubled-haploid lines derived from a cross between two Australian genotypes (RAC875 × Kukri) bred for a similar production environment. NUE field trials were carried out at four sites in South Australia and two in Western Australia across three seasons. There was genotype-by-environment-by-treatment interaction across the sites and also good transgressive segregation for yield under different N supply in the population. We detected some significant Quantitative Trait Loci (QTL) associated with NUE and N response at different rates of N application across the sites and years. It was also possible to identify lines showing positive N response based on the rankings of their Best Linear Unbiased Predictions (BLUPs) within a trial. Dissecting the complexity of the N effect on yield through QTL analysis is a key step towards elucidating the molecular and physiological basis of NUE in wheat.

## Introduction

Wheat (*Triticum aestivum* L.) is the most widely grown crop globally and a major source of carbohydrates and proteins in human nutrition. Nitrogen (N) fertilisation is critical for obtaining high grain yield (GY) and high grain protein content in this crop. The global demand for N has been increasing and was predicted to exceed 112 million tonnes in 2015, indicating the reliance of world food and fibre production on N inputs [1]. However, the increasing cost of energy is driving up the price of N fertiliser, and there are growing environmental concerns related to N pollution from runoff and leaching. The annual consumption of N fertiliser in Australian agriculture exceeds 1 million tonnes, but varies due to climate variability and price fluctuations [2]. Therefore, improving NUE in wheat, while maintaining high grain production, is an important target for breeders. NUE is also a high priority in low-yielding areas with a Mediterranean-type climate such as southern Australia. These environments are characterised by low rainfall and high temperature during late stages in the wheat growing season.

Nitrogen use efficiency (NUE) is defined as the ratio of GY to N supplied and indicates how much supplied N a plant can (i) take up (N uptake efficiency; NupE) and (ii) utilise for grain production (N utilisation efficiency; NutE) [3]. NUE and its components, NupE and NutE, are influenced by genotypic variation, environmental factors (the interaction of climate, soil, water availability and other factors) and N management [4]. Cyclic and low rainfall in low-yielding environments can intensify the side effects of excess N and result in low NUE and GY, a phenomenon known as haying-off [5]. Angus and Van Herwaarden [6] found that increased transpiration during the vegetative phase of growth (due to excessive plant vigour in response to N fertiliser) can lead to particularly inefficient water use. Increased N status can also reduce the soluble carbohydrate reserves available for re-translocation to grain after anthesis. Climate conditions, particularly rainfall amount and distribution, have an important role in N uptake and assimilation in cereals after anthesis [7]. Soil moisture is required both during and after vegetative growth to support N uptake.

To improve NUE, consideration needs to be given to genotype, environmental effects, N management and the interaction of these factors [8]. In order to improve wheat germplasm for NUE, plant breeders have assessed the genetic variation for NUE and associated traits, and G×N interaction. Previous studies revealed genetic variability for NUE, N uptake efficiency and N utilisation efficiency in maize [9], wheat [10], [11] and rice [12]. It has also been important to identify genotypes showing high NUE, but also able to yield well under both high and low N supply conditions [13]. Segregating populations made from varieties differing in N response have been used to study the genetic basis of NUE and associated traits. In a multi-environment study, Cormier *et al*. [14] assessed recent breeding progress on NUE in wheat and emphasised the value of improving NUE in varieties grown at low N supply to counteract the increasing cost of N fertiliser [15]. In addition, N management could be improved by optimising N application and synchronising crop N demand and soil N supply, reducing environmental pollution and saving money and energy [13].

QTL mapping helps provide a genetic understanding of quantitative traits and the genes controlling complex traits. Many significant QTL have been detected at high and low N in different growth conditions. For example, in wheat, An *et al*. [16], Laperche *et al*. [17] and Guo *et al*. [18] reported significant QTL in controlled conditions, and several significant genomic regions underlying NUE were detected in field trials [19], [20].

Habash *et al*. [21] undertook a QTL analysis for 21 traits related to growth, yield and leaf N assimilation during grain filling in hexaploid wheat using a mapping population from a cross between Chinese Spring and SQ1 (a high abscisic acid-expressing breeding line). They detected major QTL on chromosomes 2A, 4A and 6B for glutamine-synthetase (GS) activity, ear number per plant, peduncle N, grain N and GY. In a recent study by Xu *et al*. [22] on mapping QTL for yield and N‑related traits in wheat, regions on chromosomes 2D, 4B, 4D, 5A (2), 6A and 7A showed significant effects on N concentration in grain and shoots and NutE. Bordes *et al*. [23] identified 54 regions involving almost all chromosomes that influenced yield and its components, plant height, heading date and grain protein concentration. These chromosomal regions were proposed as good candidates to be used in breeding programs to improve the performance of wheat varieties at moderate N fertilisation rates [24], and ultimately as a resource for positional cloning of genes involved in NUE. However, the large number and variable performance of these QTL means it is unlikely breeders would actually use the information. Ideally, QTL should be identified in well-adapted germplasm and show stable performance across multiple environments or known environmental responses. The present study aimed to characterise the genetic basis of N response in a bread wheat doubled haploid (DH) population. The population used for this study was derived from a cross between two highly adapted genotypes, both bred for low-input, low-rainfall areas of Australia’s southern grain belt. Therefore, any identified QTL are of direct relevance to local breeding programs. The main objectives were to determine the genetic basis for variation in NUE for selection of N-responsive genotypes in the low-yielding environments of southern Australia.

## Materials and Methods

### Plant material

In this study we used a doubled haploid (DH) mapping population derived from a cross between the bread wheat genotypes RAC875 (female) and Kukri (male). RAC875 and Kukri have both been bred for the Mediterranean-type environment of southern Australia, but have shown marked differences in performance under severe drought and heat stress [25]. 156 DH lines from the population were planted in the South Australia (SA) trials in 2011 and 2012, and 148 DH lines in the Western Australia (WA) trials in 2013. The lines were selected from a larger DH population of 324 lines, showing similar maturity to minimise the impact of phenology on the measured traits [26].

### Field experiments

The field trials were conducted under the authority of The University of Adelaide, in South Australia and the University of Western Australia, for the Western Australian trials and adhered to the policies and practices of these two universities. A split-plot design with incomplete replication was used for all experiments. Parental lines and local genotypes as checks were always included. The local genotypes in South Australia were Correll, Drysdale, Excalibur, Frame, Gladius and Mace. In Western Australia the studied genotypes included Brookton, Bullaring, Calingiri, Datatine, Gladius, GW3118, IFlood0844, IGW2971, IGW3073, IGW3114, IGW3119, IGW3277, IGW3308, IGW3318, Mace, Machete, Reeves, Scout, Sunco, Westonia, Wyalkatchem and Yitpi. The genotypes were planted in sub-plots, with different rates of N (urea) application as the main plots (low N; no fertilisation, high N; half fertilisation and full fertilisation, depending on the usual N application practice at each site, Table 1). Soil analyses were performed by CSBP Future Farm Analytical Laboratories (Bibra Lake, Australia, Table 1). Standard regional management practices were applied to all fields and years. Heading and maturity dates, and GY (kg ha^-1^) were recorded for all plots. For each genotype, responsive grain yield (RGY) values were calculated as the difference between GY at high N and low N.

**Table 1:** The location, climate and basic soil characteristics, growing conditions and average grain yield (GY, kg ha^-1^) of five southern and western Australian trial sites used for nitrogen use efficiency field trials in this study.

Site	Year	Abbreviation	Lat^a^ (^ᵒ^ S)	Lon^b^ (^ᵒ^ E)	Elv^c^ (m)	Total rain^d^ (mm)	Hot day^e^ (d)	Soil texture^f^	pH (CaCl_2_)	pH (H_2_ O)	NH_4_^+^ nitrogen (mg kg^-1^)	NO_3_^-^ nitrogen (mg kg^-1^)	Nitrogen fertiliser levels (kg ha^-1^)	Average GY (kg ha^-1^)
**Pinery, SA**	2011	PIN 11	34.2	138.6	260	165	16	Clay	7.6	8.2	3	36	0; 75; 150	2236
**Yanco, NSW**	2011	YAN 11	34.6	146.4	164	221	22	n.a.	n.a.	n.a.	n.a.	n.a.	0;75; 150	1805
**Lameroo, SA**	2012	LAM 12	35.3	140.5	99	144	15	Loamy	8.2	9	2	8	18; 52; 87	2007
**Pinery, SA**	2012	PIN 12	34.2	138.6	260	185	23	Clay	7.7	8.5	3	54	0; 75; 150	2112
**Esperance Down, WA**	2013	ED 13	33.6	121.8	158	293	8	Loamy-sand	5.7	6.3	3	25	0; 60	3065
**Wongan Hills, WA**	2013	WH 13	30.8	116.7	305	163	26	Loamy-sand	6.5	6.9	4	22	0; 35	2559

^a^ Latitude (^ᵒ^ S)

^b^ Longitude (^ᵒ^ E)

^c^ Elevation above sea level (m)

^d^ Total rainfall during the growing season

^e^ Number of days during the growing season with a maximum temperature above 30 ^ᵒ^ C

^f^ Soil characteristics of top 10 cm depth of soil before fertilization

### Genotyping

RAC875 and Kukri population were genotyped using SNP data from an Illumina 90 K array.

Raw intensity (.idat) files for all 322 DH lines plus two replicates of the parents (RAC875 × Kukri) were imported into the polyploid version of GenomeStudio software[27] along with a custom sample-sheet and a SNP manifest file (*Wheat90k_ConsAkhunovKSU_15033654_A*.*bpm*). Prior to running the clustering algorithms within GenomeStudio, a number of quality control checks were made. Firstly, the intensity plots for measures such as signal intensity and staining controls were manually inspected in order to ensure that the intensities fell within the normal range. Secondly, low performing samples were identified by generating scatter plots, but were not excluded from the cluster calling at this stage.

Cluster patterns were generated for each SNP using a semi-automated procedure described by Wang *et al*. [27]. At the conclusion of each step, SNPs were filtered based upon metrics including call frequency and number of clusters. The filtered SNPs were then annotated following the published workflow. For example, at the conclusion of step 2, SNPs which exhibited a ‘# Clusters’ metric equal to 1 were annotated as ‘Monomorphic’. SNPs that did not fall within the criteria specified by the published workflow were assigned to a ‘No Annotation’ category. A visual examination of the cluster patterns was made and, if possible, the clusters were manually curated and the SNP annotated accordingly. From this process, there were a total of 37437 monomorphic markers, 17830 polymorphic markers and 26410 markers that exhibited multiple clusters or ambiguous cluster patterns.

### Genetic linkage map construction

Before linkage map construction, the 63757 monomorphic markers and markers with ambiguous cluster patterns were removed and the 17830 polymorphic SNP markers across the 322 DH lines were diagnostically checked. Initially, three lines containing more than 20% missing values across the marker set as well as three lines that were considered to be clones, were removed. From this reduced set, 2233 markers were removed that showed significant (*p-value* < 0.05) segregation distortion patterns that deviated from the usual 1:1 allele ratio assumed for a bi-parental population. To check the quality of the remaining SNP marker set, an initial linkage map was constructed using the MSTmap algorithm [28] integrated into the linkage map construction functions of the R/ASMap package [29] available in the R Statistical Computing Environment [30]. From this initial map the genotypes were checked across the complete genome and a total of 82 lines were removed that exhibited excessive recombination counts.

The complete set of 17830 polymorphic SNP markers for the 234 lines was then integrated with the 226 matching genotypes of the simple sequence repeat (SSR) and DArTs markers from the RAC875 × Kukri genetic linkage map described in Bennett *et al*. [24]. Prior to integration, markers in the SSR-DArTs linkage map containing more than 20% missing values were removed. The integrated SSR-DArTs-SNP marker set contained a total of 18333 markers across 226 genotypes, Marker segregation distortion was checked again and 2340 markers were removed. With the remaining 15993 markers an initial map was constructed using the MSTmap linkage map construction functions of R/ASMap. A further eight lines were removed due to excessive recombination counts, and the map was re-constructed a final time. Linkage groups with fewer than ten markers were deemed to be unlinked and omitted from further construction. Linkage group assignment and orientation was determined through a comparison of the remaining 408 SSR-DArT markers in the newly constructed linkage map with the SSR-DArT linkage map of Bennett *et al*. [25] as well as a comparison of SNP markers to the 90K SNP array based wheat consensus map. After this process, one linkage group remained unassigned, while two pairs of linkage groups and one set of three linkage groups were merged. The final integrated SSR-DArTs-SNP linkage map consisted of 218 individuals, including the all lines used in the current study, with 15911 markers assigned to 26 linkage groups. After removing co-located markers this was reduced to 1333 unique loci with a total map length of 2864.3 cM and average interval distance of 2.18 cM (minimum = 0.1 cM and maximum = 48.1 cM).

### Linear mixed model analysis

Analysis of GY was conducted using a multi-treatment-environment trial (MTET) linear mixed model that appropriately captured genetic and non-genetic sources of variation present across the multiple treatments and environments [31], [32]. For each treatment by environment the fixed component of the MTET model contained a factor that consisted of one level for the complete set of DH lines and a level for each of the parents and controls. The inclusion of this term ensured that the parents and controls remained fixed in the analysis and did not contribute to the genetic variation of the DH lines in any treatment by environment combination. In addition, for each treatment by environment combination the fixed component also contained phenology genes *ppdB1* and *ppdD1* as numerical covariates [33] as well as modelled linear trends possibly existing across the row and ranges of the environment. Extraneous non-genetic sources of design variation, such as blocks or bays, were captured using independent random effects. For each of the environment specific residuals, a separable AR1 × AR1 (AR1 = autoregressive process of order 1) process was used to adequately account for spatial correlation of GY measurements induced by the rectangular layout of the experiment.

An important component of the MTET model was the inclusion of a random effects term to model the variance-covariance structure for the genotype by treatment by environment (GTE) interaction. This structure consisted of a genetic variance of the DH lines for each treatment within an environment as well as covariances or correlations that reflect the genetic relationship of the DH lines between varying levels of N within and between environments. Due to the large number of treatment by environment combinations, this genetic random effects term was parsimoniously approximated by a Factor Analytic model [31], [32].

After fitting the MTET model, the GY BLUPs for the DH lines were extracted for all levels of N treatments within each of the environments. For any two levels of N in an environment the responsiveness GY (RGY) BLUPs for the DH lines were determined by extracting the residuals from the random regression of the GY BLUPs for the DH lines at the high level of the N treatment on the GY BLUPs for the DH lines at the low level of the treatment. The random regression line therefore represents the average performance of a DH line for the two N levels. Positive residuals from this regression indicate a genotype responded well on average to the high application of N and conversely a genotype with negative residuals indicated a poor responsiveness on average. Each two treatment combinations can then be viewed as having a GY BLUP that is equivalent to the DH line BLUPs for the lower level of the N treatment and RGY BLUP that is equivalent to the genetic response of the DH lines to the application of the higher level of the N treatment given the lower level of N.

The genetic relationships of varietal efficiency and responsiveness across trials were explored and a two-dimensional ranking system was applied to the efficiency and responsiveness of lines for all environment by treatment pair combinations. This system enabled lines to be ranked and lines having common attributes of good efficiency and responsiveness to the application of N could be selected. Similarly, the lines that showed poor efficiency and responsiveness across trials were identified. The lines were ranked in descending order based on their BLUPs in trials.

For each N treatment by environment combination broad-sense heritabilities were calculated using the formula derived in Cullis *et al*. [34]. All statistical modelling was conducted using the flexible linear mixed modelling package ASReml-R [35] available in the R statistical computing environment [30].

### QTL mapping

Using the 1333 unique loci of the integrated SSR-DArTs-SNP linkage map, QTL analyses were conducted on the GY BLUPs of the DH lines for each treatment by environment combination as well as the RGY BLUPs derived from each two level N treatment combination within each environment. The QTL analyses used the CIM approach implemented in WinQTLCart-version 2.5 (Model 6 standard analysis) [36]. LOD value thresholds were determined with 1000 fold permutations [37] and a family wise error rate *P* = 0.05. This corresponded to a minimum LOD score of 2.9. Trait abbreviations and QTL designations follow the nomenclature suggested in the wheat catalogue of gene symbols [38] with ‘asw’ signifying ‘Australian Spring Wheat’. Significant QTL were summarised with their position on a linkage group and LOD score as well as their contribution to the genetic variance.

## Results

### Grain yield analysis

**Fig 1:**
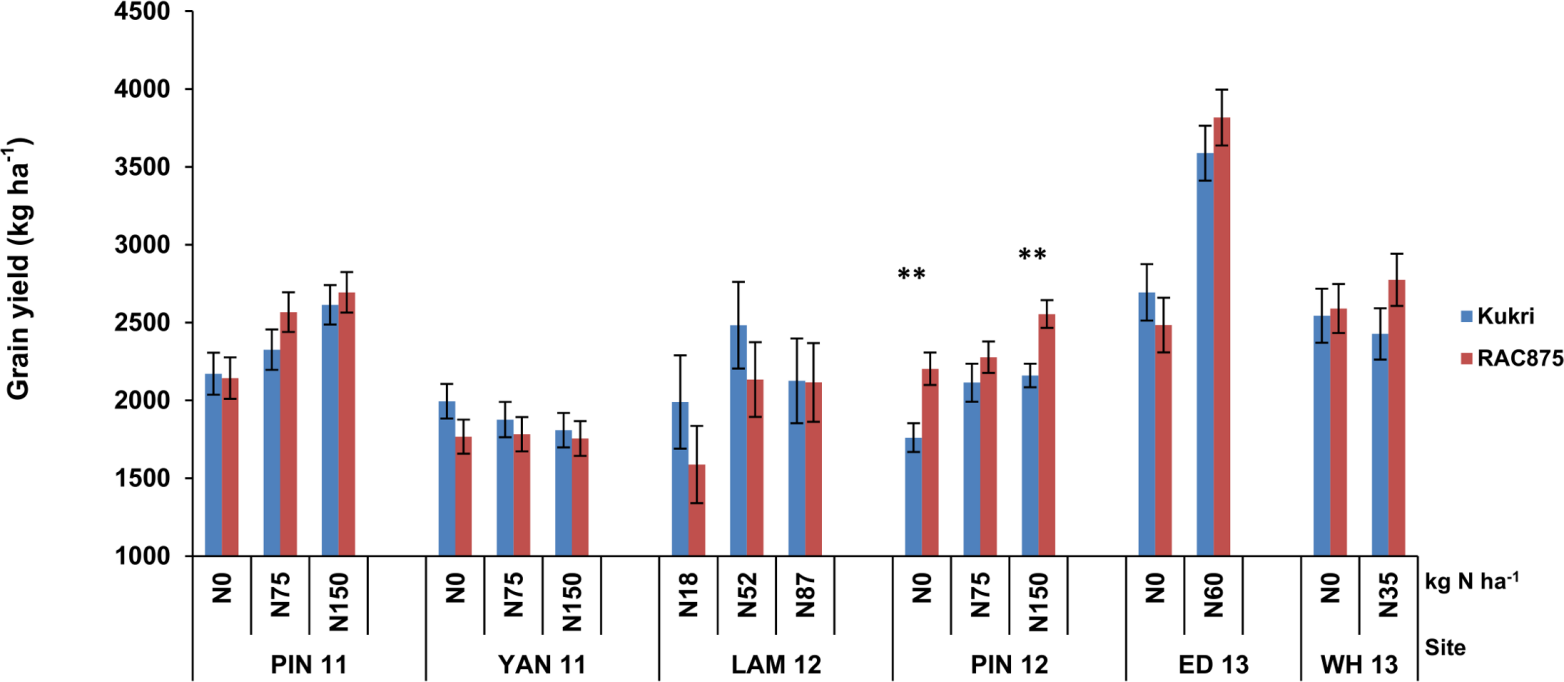
Grain yield (kg ha^-1^) of RAC875 and Kukri in six nitrogen (N) use efficiency field trials in southern Australia. The vertical error bars represent the standard errors of the predicted means after spatial analysis.

**Fig 2:**
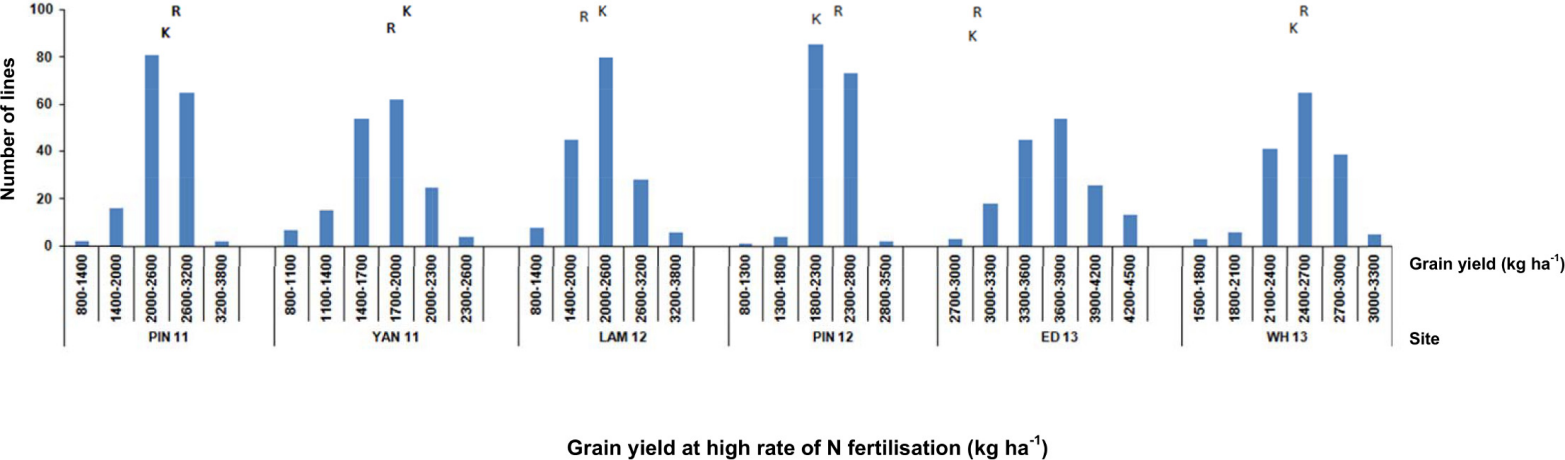
Distribution of doubled haploid lines for grain yield at high rate of nitrogen (N) fertilisation at six trial sites in South Australia and Western Australia. For each site, grain yields of RAC875 and Kukri are shown by the positions of the letters R and K, respectively.

156 and 148 RAC875 × Kukri DH lines were trialled in South Australia and Western Australia, respectively, to identify significant genetic factors underlying NUE based on GY. Average GY ranged from 1,805 kg ha^-1^ at YAN 11 to 3,065 kg ha^-1^ at ED 13 (Table 1). Under low N compared to high N conditions, yield was reduced by an average of 15% at PIN 12 and 25% at LAM 12. Parental lines showed different trends for yield performance at different N fertilisation across sites (Fig 1). For instance, the parents were significantly different for both no-fertilisation and 150 (kg N ha^-1^) levels at PIN 12, but not at other sites. In addition, at YAN 11, the parents showed no response to increasing N. Variation for GY among the DH lines exceeded the variation seen in the parental lines (Table 2 and Fig 2), demonstrating significant transgressive segregation in the population. In the initial stages of fitting the GY MTET linear mixed model we discovered there was no significant genetic variation for grain yield at 18 kg ha^-1^ of N at LAM 12. This data set was excluded from further linear mixed model analysis. The final MTET model incorporated an FA model of order 4 for the GTE interaction spanning all N treatment levels across the sites in South Australia and Western Australia.

**Table 2:** Phenotypic performance of RAC875 × Kukri population for grain yield across Australian trial sites in three seasons (2011–2013).

Site and year	Parents		DH population		
	RAC875	Kukri	Mean	Max	Min
PIN 11	2468	2370	2229	3279	593
YAN 11	1768	1893	1802	2651	795
LAM 12	1946	2200	2001	3724	739
PIN 12	2345	2012	2108	3253	871
ED 13	3151	3141	3003	4477	1472
WH 13	2683	2486	2475	3538	1316

Note: Maximum and minimum values for the population were calculated across all N fertilisation rates

The estimated genetic correlation matrix was extracted from the model and is presented in Table 3. The table indicates there are mostly moderate genetic correlations (0.40–0.70) between South Australian trial sites with stronger genetic correlations (0.71–0.99) existing between levels of N within sites. Similarly, there are also strong genetic correlations between the two levels of N within and between the Western Australian sites. Table 3 also indicates that the varying levels of N at the South Australian sites have weak or negligible genetic correlation with the two levels of N at the Western Australian sites. Broad sense heritability (the ratio of total genetic variance to total phenotypic variance) for yield in PIN 11 was highest (0.90) at 75 kg ha^-1^ N fertiliser, while it was very low at LAM 12 in all N treatments (S1 Table).

**Table 3:** Genetic correlation coefficients within sites for grain yield for all genotypes studied, parental lines and doubled haploid lines in nitrogen use efficiency field trials in Australia.

Site and year	Nitrogen Fertilisation (kg ha^-1^)	PIN 11	PIN 11	PIN 11	YAN 11	YAN 11	YAN 11	LAM 12	LAM 12	PIN 12	PIN 12	PIN 12	ED 13	ED 13	WH 13
		0	75	150	0	75	150	52	87	0	75	150	0	60	0
**PIN 11**	75	0.95													
**PIN 11**	150	0.91	0.94												
**YAN 11**	0	0.64	0.70	0.69											
**YAN 11**	75	0.68	0.73	0.73	0.99										
**YAN 11**	150	0.52	0.58	0.57	0.98	0.95									
**LAM 12**	52	0.43	0.47	0.45	0.57	0.55	0.56								
**LAM 12**	87	0.18	0.20	0.17	0.24	0.21	0.27	0.19							
**PIN 12**	0	0.39	0.36	0.35	0.42	0.39	0.57	0.27	0.22						
**PIN 12**	75	0.34	0.30	0.31	0.40	0.40	0.60	0.21	0.13	0.86					
**PIN 12**	150	0.65	0.62	0.62	0.46	0.41	0.53	0.32	0.15	0.88	0.85				
**ED 13**	0	0.14	0.13	0.06	-0.13	-0.20	-0.08	0.07	0.24	0.35	0.13	0.10			
**ED 13**	60	0.06	0.06	-0.02	-0.18	-0.26	-0.12	0.05	0.25	0.33	0.09	0.03	0.82		
**WH 13**	0	0.10	0.08	0.01	-0.33	-0.38	-0.30	-0.04	0.18	0.34	0.13	0.10	0.80	0.84	
**WH 13**	35	0.06	0.02	-0.04	-0.41	-0.45	-0.38	-0.09	0.14	0.36	0.16	0.12	0.79	0.82	0.90

### BLUPs analysis

**Fig 3:**
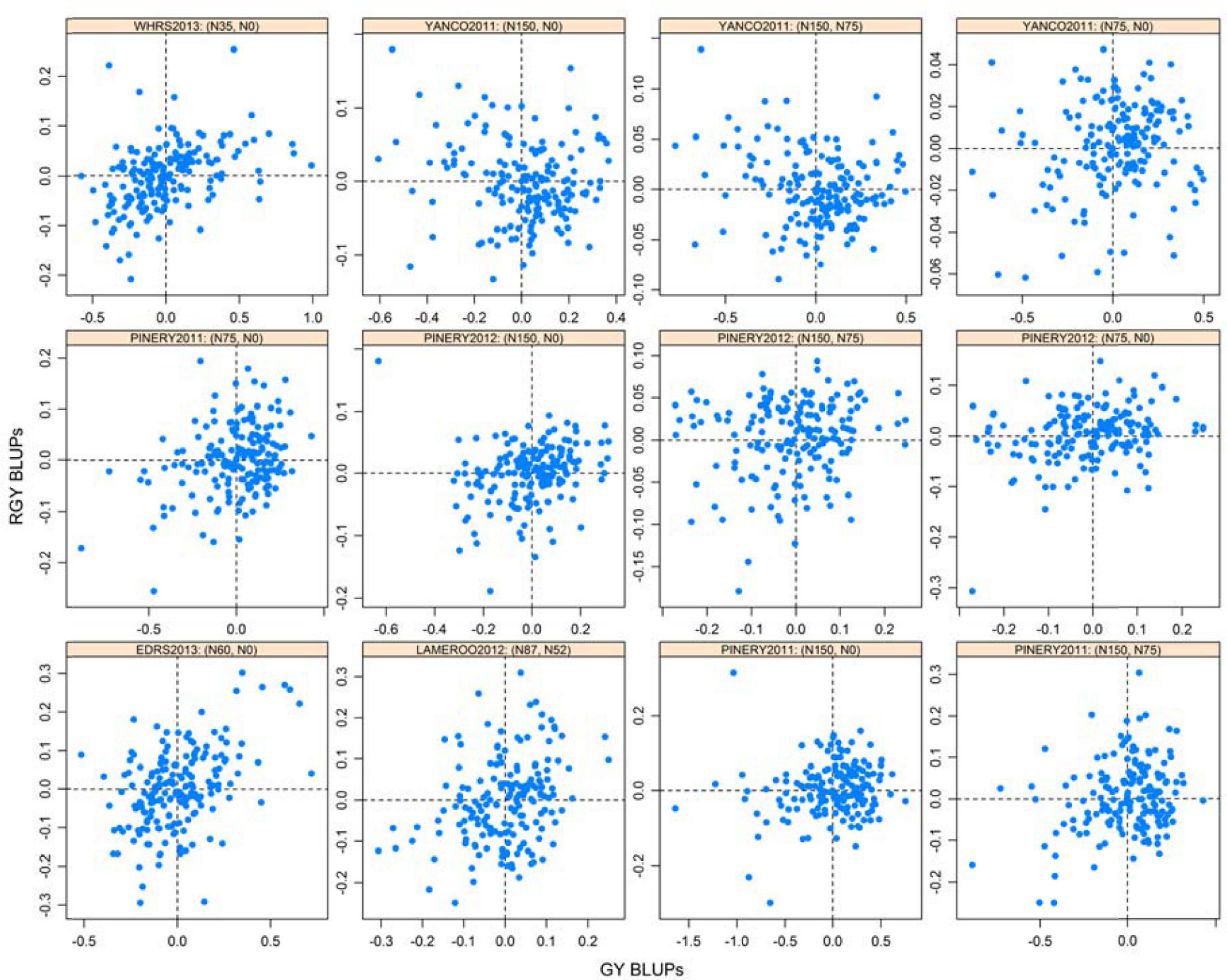
Responsive grain yield (RGY) against grain yield (GY) best linear unbiased predictions (BLUPs) of individual RAC875 × Kukri DH line across nitrogen use efficiency field trials in Australia. RAC875 and Kukri are shown by yellow and red dots, respectively.

The GY BLUPs for the DH lines were extracted from the final MTET model and RGY BLUPS for the DH lines were calculated for all N treatment combinations within each environment. For example, in PIN 12, GY BLUPs of the DH lines for N0, N75 and N150 kg ha^-1^ were extracted from the model and used to form RGY BLUPs for the DH lines denoted N75-N0, N150-N0 and N150-N75, where, for example, N150-N0 represents the response of the DH lines to the application of 150 kg ha^-1^ of N given the BLUPs for the DH lines at 0 kg N ha^-1^. Fig 3 presents a two dimensional scatter plot of the GY BLUPs for the DH lines against their RGY BLUPs for all available two N treatment combinations within an environment.

To aid interpretation, each panel is divided into four sub-areas or quadrants. The upper right quadrant (Q1) in each panel indicates DH lines that show above average GY and response to the application of N whereas those in the lower left hand quadrant (Q3) indicate below average GY and N response.

To assess the individual genetic performance of DH lines across environments and two level N treatment combinations, a two-dimensional ranking scheme was developed using the GY and RGY BLUPs (see Methods). A suitable criterion to determine the ranking of the varieties would then be based on the absolute angular differences of the varieties from these 45 degree angles as well as their lengths from the origin. The lines for good efficiency and responsiveness are ranked based on a numerical order showing consistent extension into Q1. Likewise, ranking of varieties with poor efficiency and responsiveness are in the numerical order as a consistent extension into Q3 (Fig 3).

**Fig 4:**
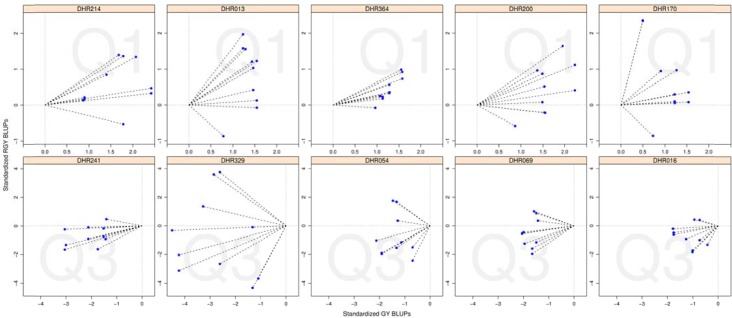
Positions of grain yield (GY) and responsive grain yield (RGY) best linear unbiased predictions (BLUPs) for all two-level nitrogen treatment combinations across the South Australian field trials, of the top five (upper row of panels; DH_R214, DH_R013, DH_R364, DH_R200, DH_R170) and bottom five (lower row of panels; DH_R241, DH_R329, DH_R054, DH_R069, DH_R016) ranked RAC875 × Kukri DH lines using a two dimensional ranking scheme. Ranking of a genotype was determined on the basis of the length of each line shown in the panels and the proximity of the lines to an optimal 45 degree angle.

Fig 4 shows the positions of the GY and RGY BLUPs for the top five (upper five panels) and bottom five (lower five panels) ranked DH lines. The length of each line and the proximity of the line to an optimal 45 degree angle provide an objective assessment of the DH genotype for each two level N treatment combination in each environment. Across all two level N treatment combinations and environments, each DH line was ranked by summing the angle differences to the optimal 45 degree line and dividing by the mean of the line lengths. Using this ranking scheme, DH_R214 was the best performing line and showed above average GY and N response in 9 of the 10 two level N treatment combinations across environments, while DH_R241 was the poorest performing line with below average GY and N response in 9 of the 10 two level N treatment combinations across environments (Fig 4).

### QTL associated with GY

In total, we identified 29 significant QTL for GY, including 17 GY-QTL on chromosomes 1A, 1B, 2A, 3D-2, 4A, 4B, 4D, 5A, 6A, 7A-1, 7B and 7D, across all treatments and environments (S1 Fig). The GY-QTL with the highest LOD score (16), additive effect and proportion of the genotypic variation explained (28%) was on chromosome 2A, for GY at high N at PIN 11 (Table 4). The only QTL that was specific for GY at low N, *QYLD*.*asw-7B*, explained only 5% of the genotypic variance. The allele from Kukri, within the interval *CAP12_c1816_325 − Kukri_c109962_396* on 7B, was responsible for an improvement in GY. There was also one QTL on 6A under high N application carrying the positive allele from RAC875 for increased GY detected only at PIN 11. The other GY-QTL were detected in both high and low N treatments, with contributions coming from both parents, showing more QTL at high N. Among the 17 GY-QTL, there were nine site-specific QTL that accounted for a relatively high proportion of the genetic variation (Table 4). These included three QTL on 4A, 4D and 7D recorded at both low and high N in YAN 11, ED 13 and PIN 12, and one on 6A at N fertilisation levels, and another one on 7B at no N. However, both Kukri (2A, 4A, 4B, 4D, 5A, 7B and 7D) and RAC875 (1A, 6A and 7A-1), along with a shared locus on 1B and two loci (one from each parent) on 3D-2 contributed to improving GY. The most significant QTL was on 2A and was recorded at four sites and all levels of N application. No QTL were detected on 2B and 2D where the *Ppd-B1* and *Ppd-D1* loci are located, confirming that the data had been adequately adjusted for these maturity effects.

**Table 4:** Genomic regions underlying the single effect of nitrogen (N) on grain yield-BLUPs, adjoining markers (closest in bold), peak position (cM), logarithm of odds (LOD), *R*^*2*^ (%) and additive effect in trials at various Australian sites.

Chr.	QTL	N treatment	Site and year	Adjoining markers	Position (cM)	LOD	*R*^*2*^ (%)	Allele effect
**1A**	1	N52	LAM 12	***Excalibur_c44711_453*** *− Excalibur_c11941_675*	17.1	2.9	6	0.02
	2	N150	PIN 11	***RAC875_rep_c104986_200*** *− RAC875_c11899_366*	43.7	5.8	9	0.1
		N0	PIN 11	*RAC875_rep_c104986_200 −* ***RAC875_c11899_366***	45.7	6.4	10	0.07
		N75	PIN 11	*RAC875_rep_c104986_200 −* ***RAC875_c11899_366***	45.7	6.7	10	0.12
		N0	YAN 11	*RAC875_rep_c104986_200 −* ***RAC875_c11899_366***	45.7	3.8	6	0.06
		N75	YAN 11	*RAC875_rep_c104986_200 −* ***RAC875_c11899_366***	45.7	4.4	7	0.05
		N0	ED 13	***RAC875_c11899_366*** *− wsnp_Ra_c20126_29372577*	46.7	3.9	8	0.05
		N60	ED 13	***RAC875_c11899_366*** *− wsnp_Ra_c20126_29372577*	46.7	3.5	7	0.07
		N35	WH 13	***RFL_Contig3715_263*** *− gwm0357*	48.9	3.7	8	0.06
**1B**	3	N75	YAN 11	*wsnp_Ex_rep_c66980_65419811 −* ***Kukri_c1529_462***	104.1	3.3	5	0.04
		N0	ED 13	***barc0207*** *− wsnp_Ex_c23992_33235984*	116.8	3.9	8	-0.05
		N60	ED 13	***barc0207*** *− wsnp_Ex_c23992_33235984*	116.8	3.6	7	-0.07
	4	N0	WH 13	*wsnp_Ex_rep_c66255_64400455 −* ***barc0256***	137	3.6	8	-0.06
		N35	WH 13	*wsnp_Ex_rep_c66255_64400455 −* ***barc0256***	137	3.9	9	-0.06
**2A**	5	N0	PIN 11	*BS00011893_51 −* ***Kukri_c46040_620***	26.7	14.4	25	-0.11
		N75	PIN 11	*BS00011893_51 −* ***Kukri_c46040_620***	26.7	15.4	27	-0.2
		N150	PIN 11	*BS00011893_51 −* ***Kukri_c46040_620***	26.7	16	28	-0.18
		N0	YAN 11	*BS00011893_51 −* ***Kukri_c46040_620***	26.7	9.8	18	-0.1
		N75	YAN 11	*BS00011893_51 −* ***Kukri_c46040_620***	26.7	11.2	19	-0.08
		N150	YAN 11	*BS00011893_51 −* ***Kukri_c46040_620***	26.7	7.7	15	-0.07
		N52	LAM 12	*BS00011893_51 −* ***Kukri_c46040_620***	26.7	5.9	12	-0.04
	6	N150	PIN 12	*D_GB5Y7FA02HSMR1_278 −* ***BobWhite_rep_c64012_389***	40.8	6.5	14	-0.04
**3D2**	7	N0	PIN 11	*cfd0064 −* ***Excalibur_c3510_1888***	18.7	5.4	8	-0.06
		N75	PIN 11	*cfd0064 −* ***Excalibur_c3510_1888***	18.7	6.9	10	-0.12
		N150	PIN 11	*cfd0064 −* ***Excalibur_c3510_1888***	18.7	6.7	10	-0.11
**3D2**		N75	YAN 11	*cfd0064 −* ***Excalibur_c3510_1888***	18.7	5.6	9	-0.06
	8	N60	ED 13	***RAC875_c35801_905*** *− wsnp_Ex_rep_c101732_87042471*	25.9	4.9	10	0.08
**4A**	9	N0	YAN 11	*Excalibur_c11047_1145 −* ***BS00064523_51***	145.4	3.4	6	-0.06
		N75	YAN 11	*Excalibur_c11047_1145 −* ***BS00064523_51***	145.4	4.5	7	-0.05
		N150	YAN 11	*Excalibur_c11047_1145 −* ***BS00064523_51***	145.4	3.7	7	-0.05
**4B**	10	N60	ED 13	*BS00004727_51 −* ***RFL_Contig5846_1610***	79.4	4	8	-0.07
		N0	WH 13	*BS00004727_51 −* ***RFL_Contig5846_1610***	79.4	4.9	11	-0.07
		N35	WH 13	*BS00068539_51 −* ***BobWhite_c4818_173***	83.1	3.7	8	-0.06
**4D**	11	N0	ED 13	*wsnp_Ex_rep_c107564_91144523 −* ***wsnp_Ku_rep_c109720_94223856***	1.8	4.4	9	-0.05
		N60	ED 13	*wsnp_Ex_rep_c79748_75305162 −* ***wsnp_BF473052D_Ta_2_1***	3.3	4.1	9	-0.07
**5A**	12	N150	YAN 11	***BS00022867_51*** *− BS00081951_51*	177.8	3.3	5	-0.04
		N0	PIN 12	***BS00022867_51*** *− BS00081951_51*	177.8	4.5	9	-0.03
		N75	PIN 12	***BS00022867_51*** *− BS00081951_51*	177.8	3.8	8	-0.04
**6A**	13	N75	PIN 11	*wsnp_Ex_c2389_4479352* ***− barc0353b***	69.5	4	6	0.09
		N150	PIN 11	*wsnp_Ex_c2389_4479352* ***− barc0353b***	67.5	4.5	7	0.09
**7A1**	14	N75	PIN 12	*wPt*.*8399* ***− Excalibur_c12996_775***	82.7	4.3	9	0.04
		N0	PIN 12	***BobWhite_rep_c49790_351 −*** *BobWhite_c16317_641*	85.1	4.5	9	0.03
	15	N0	YAN 11	*Excalibur_c49272_174* ***− wPt*.*5558***	114.4	6.1	11	0.08
		N75	YAN 11	*Excalibur_c49272_174* ***− wPt*.*5558***	114.4	4.7	7	0.05
		N150	YAN 11	*Excalibur_c49272_174* ***− wPt*.*5558***	114.4	6.2	11	0.06
		N52	LAM 12	*wPt*.*5558* ***− Ra_c114158_328***	116.4	4.2	9	0.03
**7B**	16	N0	PIN 11	*CAP12_c1816_325* ***− Kukri_c109962_396***	13.4	3.4	5	-0.05
**7D**	17	N0	PIN 12	*BobWhite_rep_c57051_479* ***− Ku_c884_1017***	76.6	4.3	9	-0.03
		N75	PIN 12	*BobWhite_rep_c57051_479* ***− Ku_c884_1017***	76.6	3.5	7	-0.04
		N150	PIN 12	***Kukri_c100613_331 −*** *RAC875_c53629_483*	83.3	4.6	9	-0.03

### QTL for N response

The responsiveness of DH lines to N application was assessed by comparing yields at different levels of N application to generate and score for RGY (Table 5). In total, 12 RGY QTLs were detected, with the predominant proportion of desirable alleles coming from the Kukri parent. These QTL were on chromosomes 1A, 1B, 2A, 2B, 3B, 3D-2 (2 loci), 5A, 6A, 6B, 7A-2 and 7B with a LOD range of 3 to 11.8. All sites revealed loci that showed a differential response to the rate of N application. Nine RGY-QTL, were classified as adaptive QTL since they were detected at only one site. These were located on chromosomes 1A, 1B, 2B, 3B, 3D-2 (2 loci), 5A, 6B and 7A-2. *QRGY*.*asw-2A* explained the highest proportion of variance (*R*^*2*^ *= 20%*) and was stable across three sites. Further, two putative QTL on 3D-2, delineated by markers *cfd0064 − Excalibur_c3510_1888* and *RAC875_rep_c79167_809 − CAP12_c1384_314*, were associated with RGY in both South Australia and Western Australia.

**Table 5:** Genomic regions underlyg the response to nitrogen (N) for grain yield-BLUPs, adjoining markers (closest in bold), peak position (cM), logarithm of odds (LOD), *R*^*2*^ (%) and additive effects in trials at various Australian sites.

Chr.	QTL	N treatment	Site and year	Adjoining markers	Position (cM)	LOD	*R*^*2*^ (%)	Allele
								effect
**1A**	1	N150-N75	PIN 12	*RAC875_rep_c104986_200 −* ***RAC875_c11899_366***	45.7	4.9	8	0.01
		N150-N0	PIN 12	*Ex_c4051_1826 −* ***wsnp_Ra_c4664_8410628***	53.9	5.3	9	0.02
**1B**	2	N150-N0	YAN 11	*Kukri_c16382_396 −* ***RAC875_c6789_838***	111.4	3.6	7	0.01
**2A**	3	N150-N75	PIN 11	*Ra_c18597_329 −* ***BS00011893_51***	17.7	4.3	9	-0.03
		N75-N0	YAN 11	***BS00011893_51*** *− Kukri_c46040_620*	19.7	4.9	10	-0.01
		N150-N0	PIN 12	*BS00011893_51 −* ***Kukri_c46040_620***	24.7	9.6	20	-0.02
		N150-N0	YAN 11	*BS00011893_51 −* ***Kukri_c46040_620***	24.7	5.1	11	0.01
		N150-N75	PIN 12	*BS00011893_51 −* ***Kukri_c46040_620***	26.7	11.8	20	-0.02
**2B**	4	N150-N0	PIN 12	***RFL_Contig3915_1042*** *− wsnp_RFL_Contig4402_5154408*	70	3.2	5	-0.01
**3B**	5	N87-N52	LAM 12	*Kukri_c32803_84 −* ***wPt*.*7984***	4.9	4	9	0.02
**3D2**	6	N150-N75	PIN 12	*cfd0064 −* ***Excalibur_c3510_1888***	18.7	4.4	7	-0.01
	7	N35-N0	WH 13	*RAC875_rep_c79167_809 −* ***CAP12_c1384_314***	79.7	3.7	10	-0.01
**5A**	8	N75-N0	PIN 12	***BS00028356_51 −*** *BS00022646_51*	154.1	3	7	-0.02
**6A**	9	N35-N0	WH 13	*wsnp_Ex_c2389_4479352 −* ***barc0353b***	69.5	4.2	10	0.01
		N60-N0	ED 13	*wsnp_Ex_c2389_4479352 −* ***barc0353b***	70.2	4.1	10	-0.02
**6B**	10	N150-N75	PIN 12	***Ex_c20409_854*** *− Ku_c2392_1692*	38.2	3.3	5	0.01
**7A2**	11	N75-N0	PIN 11	***BS00068055_51*** *− BobWhite_c23287_57*	0	3.4	8	0.02
		N150-N75	PIN 11	***BS00068055_51*** *− BobWhite_c23287_57*	0	3.4	7	0.02
**7B**	12	N150-N0	YAN 11	*wPt*.*9887 −* ***BobWhite_c25215_457***	7.1	7.1	14	0.02
		N75-N0	YAN 11	*BobWhite_c25215_457 −* ***wsnp_Ra_c3450_6434387***	7.6	5.7	11	-0.01
		N150-N75	PIN 12	*CAP12_c1816_325 −* ***Kukri_c109962_396***	13.4	5.1	8	-0.01
		N150-N0	PIN 12	*CAP12_c1816_325 −* ***Kukri_c109962_396***	13.4	4.7	8	-0.02

Several RGY regions were overlapping with QTL regions for GY. These included the regions on 1A, 1B, 2A, 3D-2, 5A, 6A and 7B (S1 Fig). Although the same regions were detected, they were not necessarily detected from the same trials; for example, the 1A RGY locus appeared in the PIN 12 trial with the same region detected in other sites for GY data. Similarly, the 3D-2, 6A and 7B RGY loci overlapped with GY-QTL from different trials (Tables 4 and 5). Two QTL were detected for GY and RGY on 3D-2, but only the locus at 18.7 cM was common. The 2A, 6A and 7B RGY loci appear to show contribution of both parents depending on the trial, but this may actually reflect two separate but closely linked loci given that the QTL peaks were slightly shifted. The RGY regions on 2B, 3B, 6B and 7A-2 were not detected in GY analysis and are therefore assumed to have no major effect on yield per se.

## Discussion

In this study GY under different rates of N application was measured across multiple sites, giving a total of 16 N×E treatments (four sites at three N rates in South Australia in 2011–2012 and two sites and two N treatments in Western Australia in 2013). The study used a population developed from two lines of bread wheat that had been bred for the same production environment, but with different genetic backgrounds. Thus, many key albeit well known adaptive traits had already been optimised in the parents (such as plant height and maturity).

An important aspect of our study was the focus on field performance in low-yielding, Mediterranean-type environments found in southern Australia. In these environments strong vegetative growth, in response to abundant N early in the growing season, can negatively impact yield due to increased water loss late in the season during flowering and grain filling [11]. Well-adapted plants are expected to be efficient in N uptake during vegetative growth, maintain optimal vegetative biomass and only mobilise N late in development. This contrasts to previous studies that have been conducted in relatively high yielding environments where large early biomass is associated with increased GY [39]. The genetic correlations were moderate to high among the South Australian sites (Table 3). This results supports the possibility of successful selection for genotypes with both high GY and NUE in these sites. However, the negative genetic correlations between some sites in the South and Western Australia suggests that transferring results across different environments can be problematic emphasising the complexity of NUE traits.

Different growth conditions in South Australia and Western Australia are likely to have caused some of the instability detected in the present QTL study. However, regions on 1A, 1B and 3D-2 for GY and also on 3D-2 for RGY were detected in both South Australia and Western Australia.

Heritability and genetic variability tended to be lowest at the low N treatment, consistent with previous studies [40], [4], [14], [41]. QTL detected on chromosomes 1A, 1B, 2A, 3D-2 and 7A-1 for GY and 2A for RGY, were producible at three or more locations and are the most stable of the 41 QTL we identified (Tables 4 and 5). These genomic regions are the best candidates for more extensive NUE studies and for positional cloning of gene(s) underlying the QTL. The remaining QTL were only detected at one or two sites. These site-specific or unstable QTL reflect regions associated with adaptation to specific environmental conditions rather than the level of applied N alone [42]. Overall maturity effects were effectively managed in these experiments by selecting lines that showed little variation for this important adaptive trait. The regions associated with variation in maturity are presented in S2 and S3 Tables.

The magnitude and direction of allelic effects across QTL showed that both parents could contribute to increased NUE and yield. This observation also helps explain the strong transgressive segregation seen across the population. Although both parents contributed desirable alleles, Kukri alleles predominated. The QTL on chromosomes 4A, 4B, 4D, 7A-1 and 7D were associated only with GY and were essentially independent of N response. Conversely, the RGY-QTL on chromosomes 2B, 3B, 6B and 7A-2 led to increased yield, making these ideal targets for enhancing NUE in improvement programs.

Some QTL detected in this study require more detailed analysis. For example, the region close to marker *RAC875_rep_c104986_200 − RAC875_c11899_366* on 1A showed a major effect on yield under low and high rates of N application at three sites, PIN 11, ED 13 and YAN 11, and is adjacent to a RGY-QTL identified at PIN 12. It seems probable that these are the same QTL observed in three separate trials. However, this needs to be verified. Importantly, these QTL regions would appear to represent a region where both N response and GY are controlled and where significant genetic gain for NUE could be achieved. In a recent study focussing on yield under drought and which used the same DH population, Bennett *et al*. [43] identified QTL for GY on chromosomes 1A, 1B, 2A, 2B, 2D, 4D, 6D and 7A. Among these QTL, regions on 1A, 1B, 2A, 4D and 7A-1 were common to the GY-QTL detected in our study, as well as a QTL on 2B for RGY. The genomic regions controlling N response were detected amongst all homoeologous chromosome groups, but the A and B genomes predominated. This observation is consistent with results found by Bogard *et al*. [44].

Many QTL for NUE and related traits have been described in wheat [15], [20], [16], [19]. Bogard *et al*. [44] detected QTL on 2D, 3B, 5A, 6B, 7A, 7B, 7D in wheat grown at various N fertilisation rates. They also found that several NUE regions co-located with QTL for grain protein content on chromosomes 2D, 3B, 5A, 7D. Similarly, Bordes *et al*. [45] found large variability in response of grain yield to N fertilisation and detected major QTL using different measures of NUE, including the difference between yield under high N versus low N (HN–LN), the ratio of yield under high N relative to low N (LN/HN) and the joint regression. They found significant regions for both GY and RGY on 1D, 2D, 3B and 5B and also for GY on 3D and for RGY on 5D. Xu *et al*. [22] found major QTL on 2D, 4B, 6A and 7A for yield components under different N supplement regimes. In their research, NUE was studied by assessing the response of GY-related traits to N fertilisation. Several of the QTL presented here are co-located with other known GY-QTL. For example, *QYLD*.*asw-1B* was detected for GY at the three sites in South Australia and West Australia. This QTL lies near to the region identified for a GY-QTL by Quarrie *et al*. [19]. Guo *et al*. [18] also reported chromosome 1B to be associated with both N uptake and utilisation in wheat.

Some of the QTL identified in these other reports are overlapping with regions identified in our own study, suggesting that some NUE-related QTL may be common to both low and high yielding environments. Interestingly, some QTL associated with N assimilation, GDH and GS activities, in wheat found by Fontain et al. [20] and Habash et al. [21] are co-located with the GY-QTL in this study. For example, coincidence of QTL for GDH activity and GY found on 2D and 5A [20] were similar to the significant regions in our study. The QTL results for both GS activity and GY on 5A and 7A [21] were in line with the presented results. These findings demonstrated the value of examining NUE components for increasing GY and the integration of physiological and molecular approaches. Novel NUE-related QTL in this study were found on 1A, 2A, 2B, 4A and 4D across various Australian sites. Identifying common genes and QTL underlying NUE traits between crops using consensus maps and meta-analyses may be useful to improve NUE in breeding programs [13].

In addition to the identification of QTL associated with GY and RGY, the present study allowed the classification of individual lines in the population based on their genetic yield and responsiveness to N fertilisation (Figs 3 and 4). The most valuable lines for breeding are those that consistently showed both a high yield and a strong response to N. In contrast to most previous studies on NUE in wheat, the parents used to develop the populations are well-adapted and commercially relevant. Consequently, their progeny are directly relevant to breeders. The most consistent high-yielding/high N response lines identified in this study have been provided to breeding programs for further development. From a research perspective, the lines that showed a consistent low N response and low yield are also of interest. These lines can be compared with the high yielding/high N response lines in biochemical and physiological studies to help determine the basis for the difference in performance and to improve screening and evaluation methods.

## Conclusion

Significant genetic variation for GY in wheat was documented at varying rates of N application. The number of QTL detected at each trial was variable, but some loci were seen across multiple trials. These loci would offer greatest benefit to breeders in selecting for improved NUE.

In addition to identifying key regions associated with NUE that could be used to track and move the desirable alleles into breeding programs, this study has identified good target regions for more detailed molecular analysis and ultimately cloning of the genes underlying the N response. The analysis allowed us to separate the relationship between yield and N supply, and also to differentiate N responsiveness of individual lines. Some QTL detected were common to both GY and RGY and will be good targets for more detailed physiological studies. Lines that show a strong response to the rate of applied N are not necessarily high yielding. However, we developed a method to rank the performance of lines both at low N and in response to N fertilisation, and identified lines that were both high yielding and highly N-responsive across multiple trials. These genotypes represent particularly attractive material for further crossing and selection given that both parents are already well-adapted for southern Australian environments.

## Supporting Information

S1 Fig Significant QTL and markers for grain yield (GY) and response to nitrogen level for GY (RGY). Distances are in cM.
(PDF)

S1 Table Heritability analysis of the sites for grain yield (GY, kg ha^-1^) at varying nitrogen (N) treatments. (DOCX)

S2 Table Genomic regions underlying the single effect of nitrogen (N) on heading date (HD), relative anthesis (RA) and relative maturity (RM), adjoining markers, peak position (cM), logarithm of odd (LOD), *R*^*2*^ (as, %) and additive allele in various Australian sites. (DOCX)

S3 Table Genomic regions underlying the response to nitrogen (N) for heading date (HD), relative anthesis (RA) and relative maturity (RM), adjoining markers, peak position (cM), logarithm of odds (LOD), *R*^*2*^ (as, %) and additive allele in various Australian sites. (DOCX)

## Abbreviations

N: Nitrogen
NUE: Nitrogen use efficiency
GY: Grain yield
RGY: Responsive grain yield
DH: Doubled haploid
QTL: Quantitative trait loci
BLUP: Best Linear Unbiased Prediction
NupE: N uptake efficiency
NutE: N utilisation efficiency
G: Genotype
SNP: Single Nucleotide Polymorphism
cM: centiMorgans
LOD: Logarithm of the Odds
CIM: Composite interval mapping
