# Supplementary material for: Genetic Basis for Variation in Wheat Grain Yield in Response to Varying Nitrogen Application
Source: PLoS One. 2016 Jul 26;11(7):e0159374. doi: 10.1371/journal.pone.0159374 (PMC4961366; doi:10.1371/journal.pone.0159374)
Supplement: S1 Fig — Distances are in cM. (PDF) [file pone.0159374.s001.pdf]

Supporting Information

Genetic Basis for Variation in Wheat Grain Yield in Response to Varying Nitrogen Application

Saba Mahjourimajd[1], Julian Taylor [3], Beata Sznajder[1], Andy Timmins[1], Fahimeh Shahinnia[1, #a], Zed Rengel[4], Hossein Khabaz-Saberi[4], Haydn Kuchel[2], Mamoru Okamoto[1*], Peter Langridge[1*]

[1]Australian Centre for Plant Functional Genomics (ACPFG), The University of Adelaide, PMB1, Glen Osmond, SA 5064, Australia

[2]Australian Grain Technologies, PMB1, Glen Osmond, SA 5064, Australia

[3]School of Agriculture, Food and Wine, Waite Research Institute, The University of Adelaide, PMB 1, Glen Osmond, SA 5064, Australia

[4]Soil Science and Plant Nutrition M087, School of Earth and Environment, University of Western Australia, 35 Stirling Highway, Crawley WA 6009, Australia

Present address:

[#a]Current Address: Leibniz-Institute of Plant Genetics and Crop Plant Research (IPK), Corrensstr. 3, 06466 Gatersleben, Germany

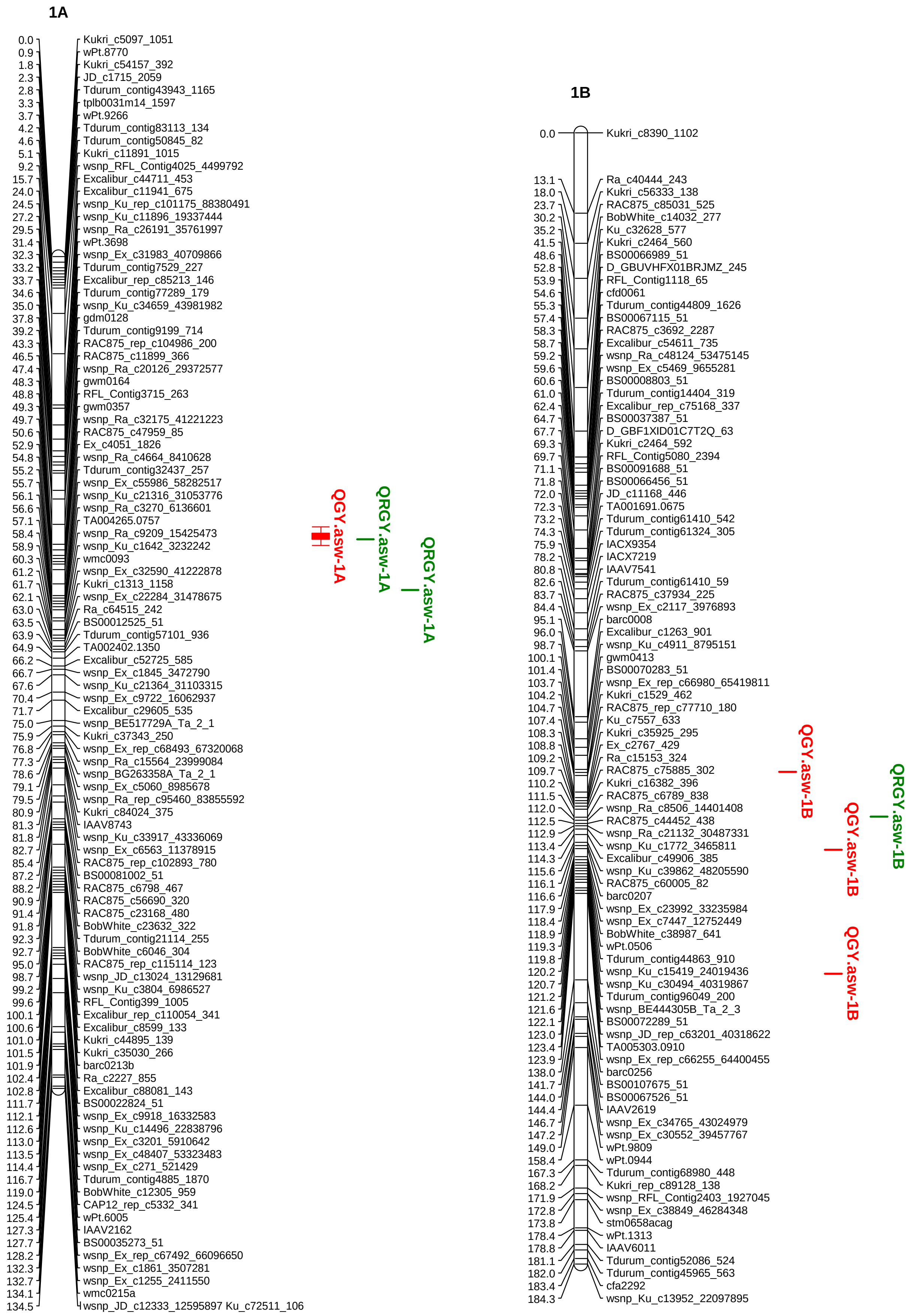

S1 Fig. Significant QTL and markers for grain yield (GY) and response to nitrogen level for GY (RGY). Distances are in cM

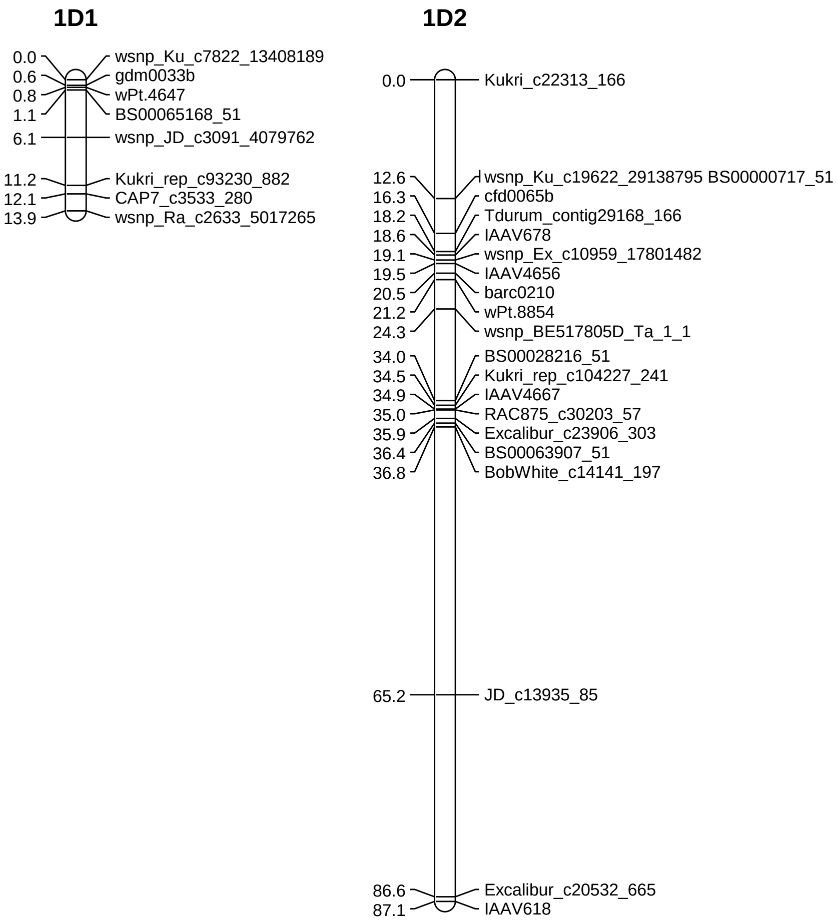
1D1
0.0 wsnp_Ku_c7822_13408189
0.6 gdm0033b
0.8 wPt.4647
1.1 BS00065168_51
6.1 wsnp_JD_c3091_4079762
11.2 Kukri_rep_c93230_882
12.1 CAP7_c3533_280
13.9 wsnp_Ra_c2633_5017265
1D2
0.0 Kukri_c22313_166
12.6 wsnp_Ku_c19622_29138795 BS00000717_51
16.3 cfd0065b
18.2 Tdurum_contig29168_166
18.6 IAAV678
19.1 wsnp_Ex_c10959_17801482
19.5 IAAV4656
20.5 barc0210
21.2 wPt.8854
24.3 wsnp_BE517805D_Ta_1_1
34.0 BS00028216_51
34.5 Kukri_rep_c104227_241
34.9 IAAV4667
35.0 RAC875_c30203_57
35.9 Excalibur_c23906_303
36.4 BS00063907_51
36.8 BobWhite_c14141_197
65.2 JD_c13935_85
86.6 Excalibur_c20532_665
87.1 IAAV618

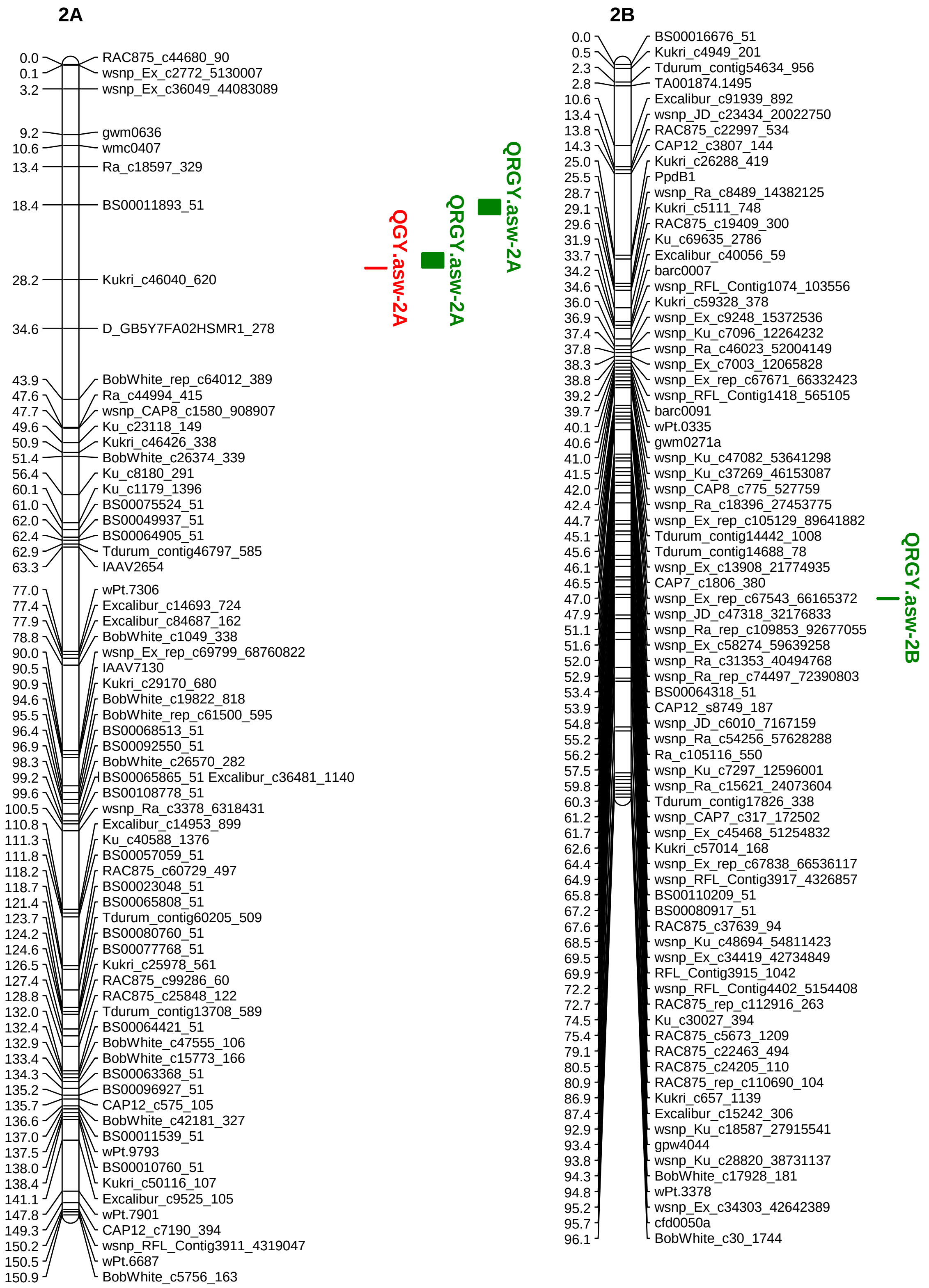
2A
0.0 RAC875_c44680_90
0.1 wsnp_Ex_c2772_5130007
3.2 wsnp_Ex_c36049_44083089
9.2 gwm0636
10.6 wmc0407
13.4 Ra_c18597_329
18.4 BS00011893_51
28.2 Kukri_c46040_620
34.6 D_GB5Y7FA02HSMR1_278
43.9 BobWhite_rep_c64012_389
47.6 Ra_c44994_415
47.7 wsnp_CAP8_c1580_908907
49.6 Ku_c23118_149
50.9 Kukri_c46426_338
51.4 BobWhite_c26374_339
56.4 Ku_c8180_291
60.1 Ku_c1179_1396
61.0 BS00075524_51
62.0 BS00049937_51
62.4 BS00064905_51
62.9 Tdurum_contig46797_585
63.3 IAAV2654
77.0 wPt.7306
77.4 Excalibur_c14693_724
77.9 Excalibur_c84687_162
78.8 BobWhite_c1049_338
90.0 wsnp_Ex_rep_c69799_68760822
90.5 IAAV7130
90.9 Kukri_c29170_680
94.6 BobWhite_c19822_818
95.5 BobWhite_rep_c61500_595
96.4 BS00068513_51
96.9 BS00092550_51
98.3 BobWhite_c26570_282
99.2 BS00065865_51 Excalibur_c36481_1140
99.6 BS00108778_51
100.5 wsnp_Ra_c3378_6318431
110.8 Excalibur_c14953_899
111.3 Ku_c40588_1376
111.8 BS00057059_51
118.2 RAC875_c60729_497
118.7 BS00023048_51
121.4 BS00065808_51
123.7 Tdurum_contig60205_509
124.2 BS00080760_51
124.6 BS00077768_51
126.5 Kukri_c25978_561
127.4 RAC875_c99286_60
128.8 RAC875_c25848_122
132.0 Tdurum_contig13708_589
132.4 BS00064421_51
132.9 BobWhite_c47555_106
133.4 BobWhite_c15773_166
134.3 BS00063368_51
135.2 BS00096927_51
135.7 CAP12_c575_105
136.6 BobWhite_c42181_327
137.0 BS00011539_51
137.5 wPt.9793
138.0 BS00010760_51
138.4 Kukri_c50116_107
141.1 Excalibur_c9525_105
147.8 wPt.7901
149.3 CAP12_c7190_394
150.2 wsnp_RFL_Contig3911_4319047
150.5 wPt.6687
150.9 BobWhite_c5756_163
QGY.asw-2A
QRGY.asw-2A
QRGY.asw-2A
2B
0.0 BS00016676_51
0.5 Kukri_c4949_201
2.3 Tdurum_contig54634_956
2.8 TA001874.1495
10.6 Excalibur_c91939_892
13.4 wsnp_JD_c23434_20022750
13.8 RAC875_c22997_534
14.3 CAP12_c3807_144
25.0 Kukri_c26288_419
25.5 PpdB1
28.7 wsnp_Ra_c8489_14382125
29.1 Kukri_c5111_748
29.6 RAC875_c19409_300
31.9 Ku_c69635_2786
33.7 Excalibur_c40056_59
34.2 barc0007
34.6 wsnp_RFL_Contig1074_103556
36.0 Kukri_c59328_378
36.9 wsnp_Ex_c9248_15372536
37.4 wsnp_Ku_c7096_12264232
37.8 wsnp_Ra_c46023_52004149
38.3 wsnp_Ex_c7003_12065828
38.8 wsnp_Ex_rep_c67671_66332423
39.2 wsnp_RFL_Contig1418_565105
39.7 barc0091
40.1 wPt.0335
40.6 gwm0271a
41.0 wsnp_Ku_c47082_53641298
41.5 wsnp_Ku_c37269_46153087
42.0 wsnp_CAP8_c775_527759
42.4 wsnp_Ra_c18396_27453775
44.7 wsnp_Ex_rep_c105129_89641882
45.1 Tdurum_contig14442_1008
45.6 Tdurum_contig14688_78
46.1 wsnp_Ex_c13908_21774935
46.5 CAP7_c1806_380
47.0 wsnp_Ex_rep_c67543_66165372
47.9 wsnp_JD_c47318_32176833
51.1 wsnp_Ra_rep_c109853_92677055
51.6 wsnp_Ex_c58274_59639258
52.0 wsnp_Ra_c31353_40494768
52.9 wsnp_Ra_rep_c74497_72390803
53.4 BS00064318_51
53.9 CAP12_s8749_187
54.8 wsnp_JD_c6010_7167159
55.2 wsnp_Ra_c54256_57628288
56.2 Ra_c105116_550
57.5 wsnp_Ku_c7297_12596001
59.8 wsnp_Ra_c15621_24073604
60.3 Tdurum_contig17826_338
61.2 wsnp_CAP7_c317_172502
61.7 wsnp_Ex_c45468_51254832
62.6 Kukri_c57014_168
64.4 wsnp_Ex_rep_c67838_66536117
64.9 wsnp_RFL_Contig3917_4326857
65.8 BS00110209_51
67.2 BS00080917_51
67.6 RAC875_c37639_94
68.5 wsnp_Ku_c48694_54811423
69.5 wsnp_Ex_c34419_42734849
69.9 RFL_Contig3915_1042
72.2 wsnp_RFL_Contig4402_5154408
72.7 RAC875_rep_c112916_263
74.5 Ku_c30027_394
75.4 RAC875_c5673_1209
79.1 RAC875_c22463_494
80.5 RAC875_c24205_110
80.9 RAC875_rep_c110690_104
86.9 Kukri_c657_1139
87.4 Excalibur_c15242_306
92.9 wsnp_Ku_c18587_27915541
93.4 gpw4044
93.8 wsnp_Ku_c28820_38731137
94.3 BobWhite_c17928_181
94.8 wPt.3378
95.2 wsnp_Ex_c34303_42642389
95.7 cfd0050a
96.1 BobWhite_c30_1744
QRGY.asw-2B

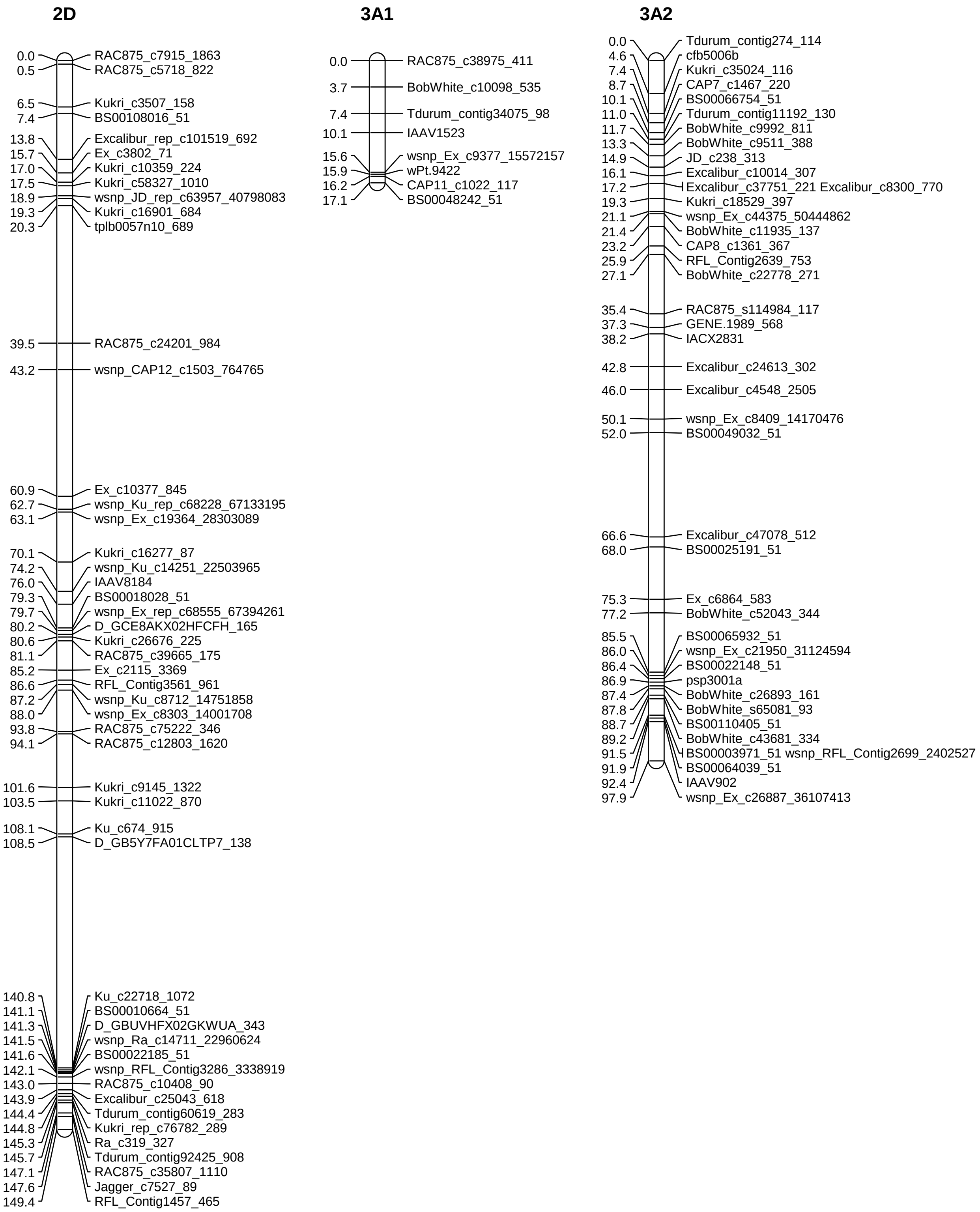
2D
0.0 RAC875_c7915_1863
0.5 RAC875_c5718_822
6.5 Kukri_c3507_158
7.4 BS00108016_51
13.8 Excalibur_rep_c101519_692
15.7 Ex_c3802_71
17.0 Kukri_c10359_224
17.5 Kukri_c58327_1010
18.9 wsnp_JD_rep_c63957_40798083
19.3 Kukri_c16901_684
20.3 tplb0057n10_689
39.5 RAC875_c24201_984
43.2 wsnp_CAP12_c1503_764765
60.9 Ex_c10377_845
62.7 wsnp_Ku_rep_c68228_67133195
63.1 wsnp_Ex_c19364_28303089
70.1 Kukri_c16277_87
74.2 wsnp_Ku_c14251_22503965
76.0 IAAV8184
79.3 BS00018028_51
79.7 wsnp_Ex_rep_c68555_67394261
80.2 D_GCE8AKX02HFCFH_165
80.6 Kukri_c26676_225
81.1 RAC875_c39665_175
85.2 Ex_c2115_3369
86.6 RFL_Contig3561_961
87.2 wsnp_Ku_c8712_14751858
88.0 wsnp_Ex_c8303_14001708
93.8 RAC875_c75222_346
94.1 RAC875_c12803_1620
101.6 Kukri_c9145_1322
103.5 Kukri_c11022_870
108.1 Ku_c674_915
108.5 D_GB5Y7FA01CLTP7_138
140.8 Ku_c22718_1072
141.1 BS00010664_51
141.3 D_GBUVHFX02GKWUA_343
141.5 wsnp_Ra_c14711_22960624
141.6 BS00022185_51
142.1 wsnp_RFL_Contig3286_3338919
143.0 RAC875_c10408_90
143.9 Excalibur_c25043_618
144.4 Tdurum_contig60619_283
144.8 Kukri_rep_c76782_289
145.3 Ra_c319_327
145.7 Tdurum_contig92425_908
147.1 RAC875_c35807_1110
147.6 Jagger_c7527_89
149.4 RFL_Contig1457_465
3A1
0.0 RAC875_c38975_411
3.7 BobWhite_c10098_535
7.4 Tdurum_contig34075_98
10.1 IAAV1523
15.6 wsnp_Ex_c9377_15572157
15.9 wPt.9422
16.2 CAP11_c1022_117
17.1 BS00048242_51
3A2
0.0 Tdurum_contig274_114
4.6 cfb5006b
7.4 Kukri_c35024_116
8.7 CAP7_c1467_220
10.1 BS00066754_51
11.0 Tdurum_contig11192_130
11.7 BobWhite_c9992_811
13.3 BobWhite_c9511_388
14.9 JD_c238_313
16.1 Excalibur_c10014_307
17.2 Excalibur_c37751_221 Excalibur_c8300_770
19.3 Kukri_c18529_397
21.1 wsnp_Ex_c44375_50444862
21.4 BobWhite_c11935_137
23.2 CAP8_c1361_367
25.9 RFL_Contig2639_753
27.1 BobWhite_c22778_271
35.4 RAC875_s114984_117
37.3 GENE.1989_568
38.2 IACX2831
42.8 Excalibur_c24613_302
46.0 Excalibur_c4548_2505
50.1 wsnp_Ex_c8409_14170476
52.0 BS00049032_51
66.6 Excalibur_c47078_512
68.0 BS00025191_51
75.3 Ex_c6864_583
77.2 BobWhite_c52043_344
85.5 BS00065932_51
86.0 wsnp_Ex_c21950_31124594
86.4 BS00022148_51
86.9 psp3001a
87.4 BobWhite_c26893_161
87.8 BobWhite_s65081_93
88.7 BS00110405_51
89.2 BobWhite_c43681_334
91.5 BS00003971_51 wsnp_RFL_Contig2699_2402527
91.9 BS00064039_51
92.4 IAAV902
97.9 wsnp_Ex_c26887_36107413

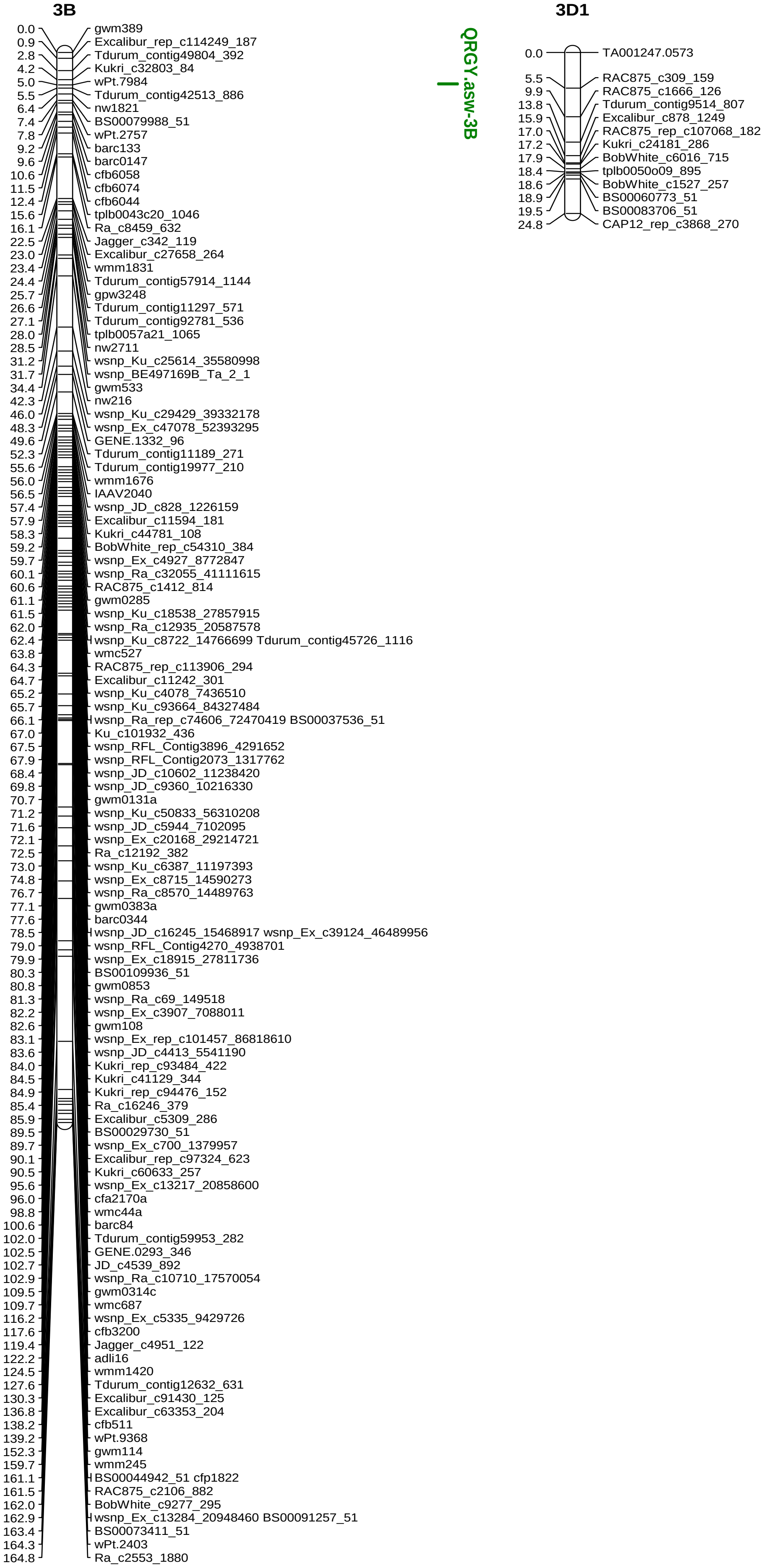
3B
0.0 gwm389
0.9 Excalibur_rep_c114249_187
2.8 Tdurum_contig49804_392
4.2 Kukri_c32803_84
5.0 wPt.7984
5.5 Tdurum_contig42513_886
6.4 nw1821
7.4 BS00079988_51
7.8 wPt.2757
9.2 barc133
9.6 barc0147
10.6 cfb6058
11.5 cfb6074
12.4 cfb6044
15.6 tplb0043c20_1046
16.1 Ra_c8459_632
22.5 Jagger_c342_119
23.0 Excalibur_c27658_264
23.4 wmm1831
24.4 Tdurum_contig57914_1144
25.7 gpw3248
26.6 Tdurum_contig11297_571
27.1 Tdurum_contig92781_536
28.0 tplb0057a21_1065
28.5 nw2711
31.2 wsnp_Ku_c25614_35580998
31.7 wsnp_BE497169B_Ta_2_1
34.4 gwm533
42.3 nw216
46.0 wsnp_Ku_c29429_39332178
48.3 wsnp_Ex_c47078_52393295
49.6 GENE.1332_96
52.3 Tdurum_contig11189_271
55.6 Tdurum_contig19977_210
56.0 wmm1676
56.5 IAAV2040
57.4 wsnp_JD_c828_1226159
57.9 Excalibur_c11594_181
58.3 Kukri_c44781_108
59.2 BobWhite_rep_c54310_384
59.7 wsnp_Ex_c4927_8772847
60.1 wsnp_Ra_c32055_41111615
60.6 RAC875_c1412_814
61.1 gwm0285
61.5 wsnp_Ku_c18538_27857915
62.0 wsnp_Ra_c12935_20587578
62.4 wsnp_Ku_c8722_14766699 Tdurum_contig45726_1116
63.8 wmc527
64.3 RAC875_rep_c113906_294
64.7 Excalibur_c11242_301
65.2 wsnp_Ku_c4078_7436510
65.7 wsnp_Ku_c93664_84327484
66.1 wsnp_Ra_rep_c74606_72470419 BS00037536_51
67.0 Ku_c101932_436
67.5 wsnp_RFL_Contig3896_4291652
67.9 wsnp_RFL_Contig2073_1317762
68.4 wsnp_JD_c10602_11238420
69.8 wsnp_JD_c9360_10216330
70.7 gwm0131a
71.2 wsnp_Ku_c50833_56310208
71.6 wsnp_JD_c5944_7102095
72.1 wsnp_Ex_c20168_29214721
72.5 Ra_c12192_382
73.0 wsnp_Ku_c6387_11197393
74.8 wsnp_Ex_c8715_14590273
76.7 wsnp_Ra_c8570_14489763
77.1 gwm0383a
77.6 barc0344
78.5 wsnp_JD_c16245_15468917 wsnp_Ex_c39124_46489956
79.0 wsnp_RFL_Contig4270_4938701
79.9 wsnp_Ex_c18915_27811736
80.3 BS00109936_51
80.8 gwm0853
81.3 wsnp_Ra_c69_149518
82.2 wsnp_Ex_c3907_7088011
82.6 gwm108
83.1 wsnp_Ex_rep_c101457_86818610
83.6 wsnp_JD_c4413_5541190
84.0 Kukri_rep_c93484_422
84.5 Kukri_c41129_344
84.9 Kukri_rep_c94476_152
85.4 Ra_c16246_379
85.9 Excalibur_c5309_286
89.5 BS00029730_51
89.7 wsnp_Ex_c700_1379957
90.1 Excalibur_rep_c97324_623
90.5 Kukri_c60633_257
95.6 wsnp_Ex_c13217_20858600
96.0 cfa2170a
98.8 wmc44a
100.6 barc84
102.0 Tdurum_contig59953_282
102.5 GENE.0293_346
102.7 JD_c4539_892
102.9 wsnp_Ra_c10710_17570054
109.5 gwm0314c
109.7 wmc687
116.2 wsnp_Ex_c5335_9429726
117.6 cfb3200
119.4 Jagger_c4951_122
122.2 adli16
124.5 wmm1420
127.6 Tdurum_contig12632_631
130.3 Excalibur_c91430_125
136.8 Excalibur_c63353_204
138.2 cfb511
139.2 wPt.9368
152.3 gwm114
159.7 wmm245
161.1 BS00044942_51 cfp1822
161.5 RAC875_c2106_882
162.0 BobWhite_c9277_295
162.9 wsnp_Ex_c13284_20948460 BS00091257_51
163.4 BS00073411_51
164.3 wPt.2403
164.8 Ra_c2553_1880
QRGY.asw-3B
3D1
0.0 TA001247.0573
5.5 RAC875_c309_159
9.9 RAC875_c1666_126
13.8 Tdurum_contig9514_807
15.9 Excalibur_c878_1249
17.0 RAC875_rep_c107068_182
17.2 Kukri_c24181_286
17.9 BobWhite_c6016_715
18.4 tplb0050o09_895
18.6 BobWhite_c1527_257
18.9 BS00060773_51
19.5 BS00083706_51
24.8 CAP12_rep_c3868_270

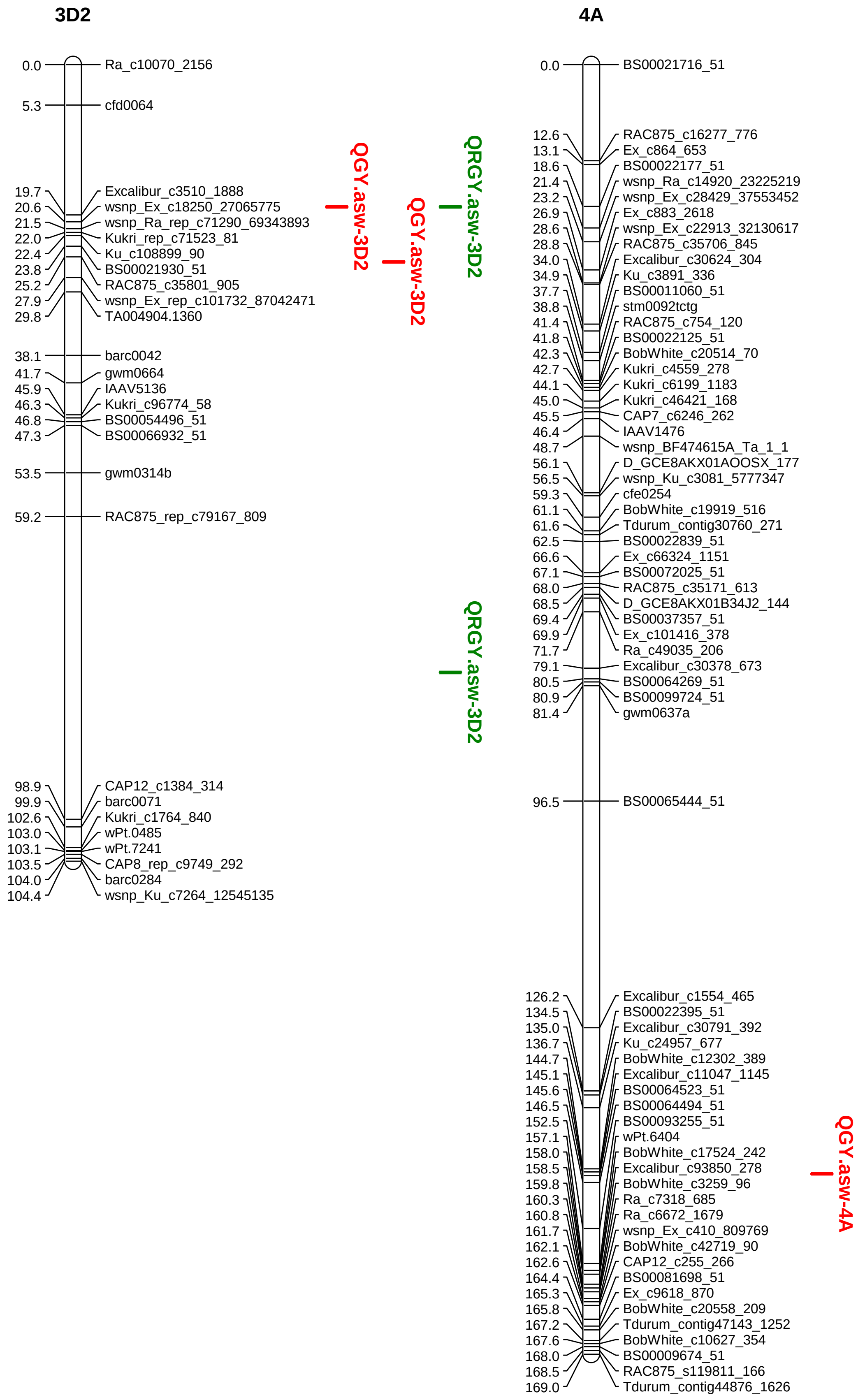
3D2
4A
0.0 Ra_c10070_2156
5.3 cfd0064
19.7 Excalibur_c3510_1888
20.6 wsnp_Ex_c18250_27065775
21.5 wsnp_Ra_rep_c71290_69343893
22.0 Kukri_rep_c71523_81
22.4 Ku_c108899_90
23.8 BS00021930_51
25.2 RAC875_c35801_905
27.9 wsnp_Ex_rep_c101732_87042471
29.8 TA004904.1360
38.1 barc0042
41.7 gwm0664
45.9 IAAV5136
46.3 Kukri_c96774_58
46.8 BS00054496_51
47.3 BS00066932_51
53.5 gwm0314b
59.2 RAC875_rep_c79167_809
98.9 CAP12_c1384_314
99.9 barc0071
102.6 Kukri_c1764_840
103.0 wPt.0485
103.1 wPt.7241
103.5 CAP8_rep_c9749_292
104.0 barc0284
104.4 wsnp_Ku_c7264_12545135
QGY.asw-3D2
QGY.asw-3D2
QRGY.asw-3D2
QRGY.asw-3D2
0.0 BS00021716_51
12.6 RAC875_c16277_776
13.1 Ex_c864_653
18.6 BS00022177_51
21.4 wsnp_Ra_c14920_23225219
23.2 wsnp_Ex_c28429_37553452
26.9 Ex_c883_2618
28.6 wsnp_Ex_c22913_32130617
28.8 RAC875_c35706_845
34.0 Excalibur_c30624_304
34.9 Ku_c3891_336
37.7 BS00011060_51
38.8 stm0092tctg
41.4 RAC875_c754_120
41.8 BS00022125_51
42.3 BobWhite_c20514_70
42.7 Kukri_c4559_278
44.1 Kukri_c6199_1183
45.0 Kukri_c46421_168
45.5 CAP7_c6246_262
46.4 IAAV1476
48.7 wsnp_BF474615A_Ta_1_1
56.1 D_GCE8AKX01AOOSX_177
56.5 wsnp_Ku_c3081_5777347
59.3 cfe0254
61.1 BobWhite_c19919_516
61.6 Tdurum_contig30760_271
62.5 BS00022839_51
66.6 Ex_c66324_1151
67.1 BS00072025_51
68.0 RAC875_c35171_613
68.5 D_GCE8AKX01B34J2_144
69.4 BS00037357_51
69.9 Ex_c101416_378
71.7 Ra_c49035_206
79.1 Excalibur_c30378_673
80.5 BS00064269_51
80.9 BS00099724_51
81.4 gwm0637a
96.5 BS00065444_51
126.2 Excalibur_c1554_465
134.5 BS00022395_51
135.0 Excalibur_c30791_392
136.7 Ku_c24957_677
144.7 BobWhite_c12302_389
145.1 Excalibur_c11047_1145
145.6 BS00064523_51
146.5 BS00064494_51
152.5 BS00093255_51
157.1 wPt.6404
158.0 BobWhite_c17524_242
158.5 Excalibur_c93850_278
159.8 BobWhite_c3259_96
160.3 Ra_c7318_685
160.8 Ra_c6672_1679
161.7 wsnp_Ex_c410_809769
162.1 BobWhite_c42719_90
162.6 CAP12_c255_266
164.4 BS00081698_51
165.3 Ex_c9618_870
165.8 BobWhite_c20558_209
167.2 Tdurum_contig47143_1252
167.6 BobWhite_c10627_354
168.0 BS00009674_51
168.5 RAC875_s119811_166
169.0 Tdurum_contig44876_1626
QGY.asw-4A

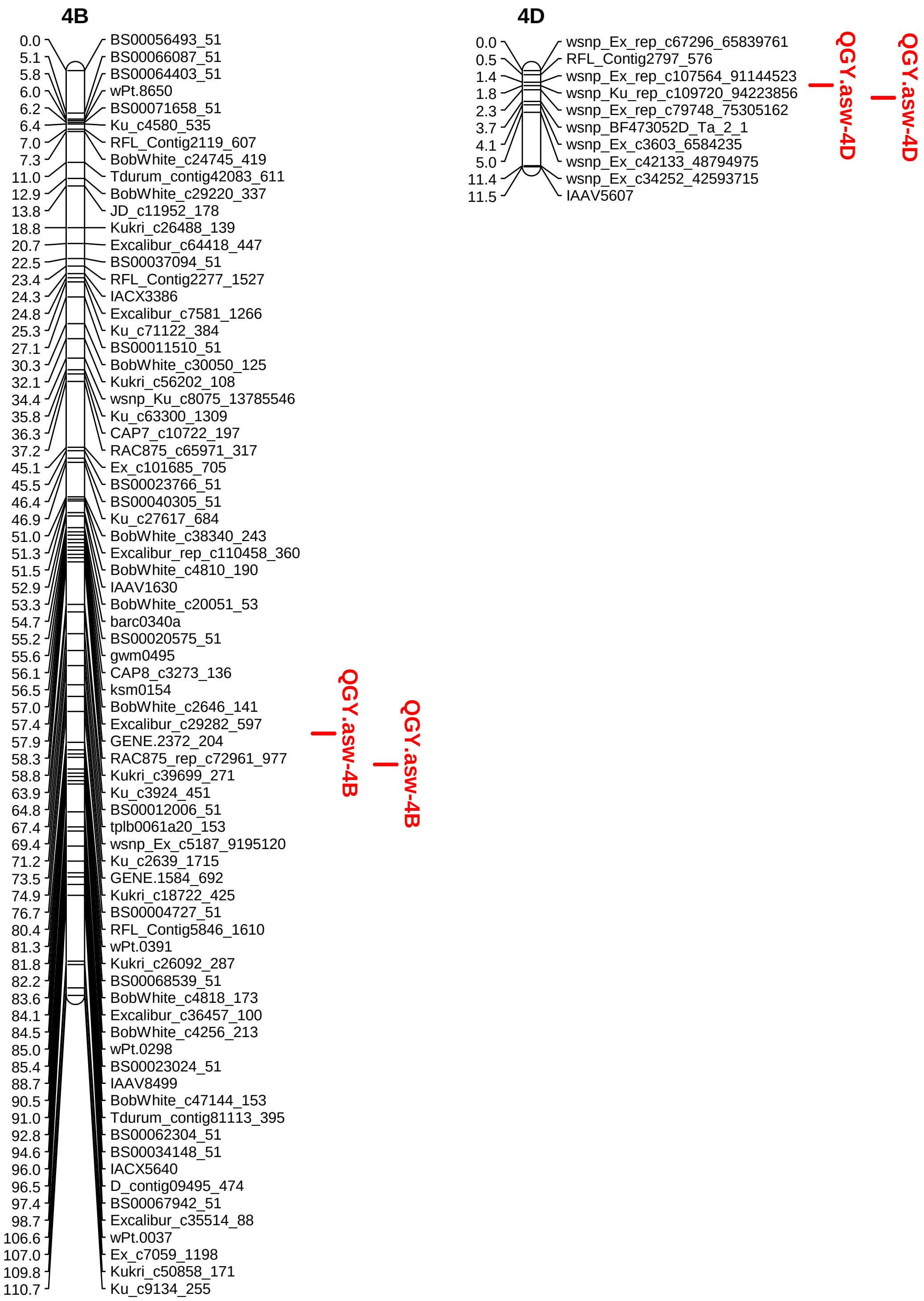
4B
0.0 BS00056493_51
5.1 BS00066087_51
5.8 BS00064403_51
6.0 wPt.8650
6.2 BS00071658_51
6.4 Ku_c4580_535
7.0 RFL_Contig2119_607
7.3 BobWhite_c24745_419
11.0 Tdurum_contig42083_611
12.9 BobWhite_c29220_337
13.8 JD_c11952_178
18.8 Kukri_c26488_139
20.7 Excalibur_c64418_447
22.5 BS00037094_51
23.4 RFL_Contig2277_1527
24.3 IACX3386
24.8 Excalibur_c7581_1266
25.3 Ku_c71122_384
27.1 BS00011510_51
30.3 BobWhite_c30050_125
32.1 Kukri_c56202_108
34.4 wsnp_Ku_c8075_13785546
35.8 Ku_c63300_1309
36.3 CAP7_c10722_197
37.2 RAC875_c65971_317
45.1 Ex_c101685_705
45.5 BS00023766_51
46.4 BS00040305_51
46.9 Ku_c27617_684
51.0 BobWhite_c38340_243
51.3 Excalibur_rep_c110458_360
51.5 BobWhite_c4810_190
52.9 IAAV1630
53.3 BobWhite_c20051_53
54.7 barc0340a
55.2 BS00020575_51
55.6 gwm0495
56.1 CAP8_c3273_136
56.5 ksm0154
57.0 BobWhite_c2646_141
57.4 Excalibur_c29282_597
57.9 GENE.2372_204
58.3 RAC875_rep_c72961_977
58.8 Kukri_c39699_271
63.9 Ku_c3924_451
64.8 BS00012006_51
67.4 tplb0061a20_153
69.4 wsnp_Ex_c5187_9195120
71.2 Ku_c2639_1715
73.5 GENE.1584_692
74.9 Kukri_c18722_425
76.7 BS00004727_51
80.4 RFL_Contig5846_1610
81.3 wPt.0391
81.8 Kukri_c26092_287
82.2 BS00068539_51
83.6 BobWhite_c4818_173
84.1 Excalibur_c36457_100
84.5 BobWhite_c4256_213
85.0 wPt.0298
85.4 BS00023024_51
88.7 IAAV8499
90.5 BobWhite_c47144_153
91.0 Tdurum_contig81113_395
92.8 BS00062304_51
94.6 BS00034148_51
96.0 IACX5640
96.5 D_contig09495_474
97.4 BS00067942_51
98.7 Excalibur_c35514_88
106.6 wPt.0037
107.0 Ex_c7059_1198
109.8 Kukri_c50858_171
110.7 Ku_c9134_255
QGY.asw-4B
QGY.asw-4B
4D
0.0 wsnp_Ex_rep_c67296_65839761
0.5 RFL_Contig2797_576
1.4 wsnp_Ex_rep_c107564_91144523
1.8 wsnp_Ku_rep_c109720_94223856
2.3 wsnp_Ex_rep_c79748_75305162
3.7 wsnp_BF473052D_Ta_2_1
4.1 wsnp_Ex_c3603_6584235
5.0 wsnp_Ex_c42133_48794975
11.4 wsnp_Ex_c34252_42593715
11.5 IAAV5607
QGY.asw-4D
QGY.asw-4D

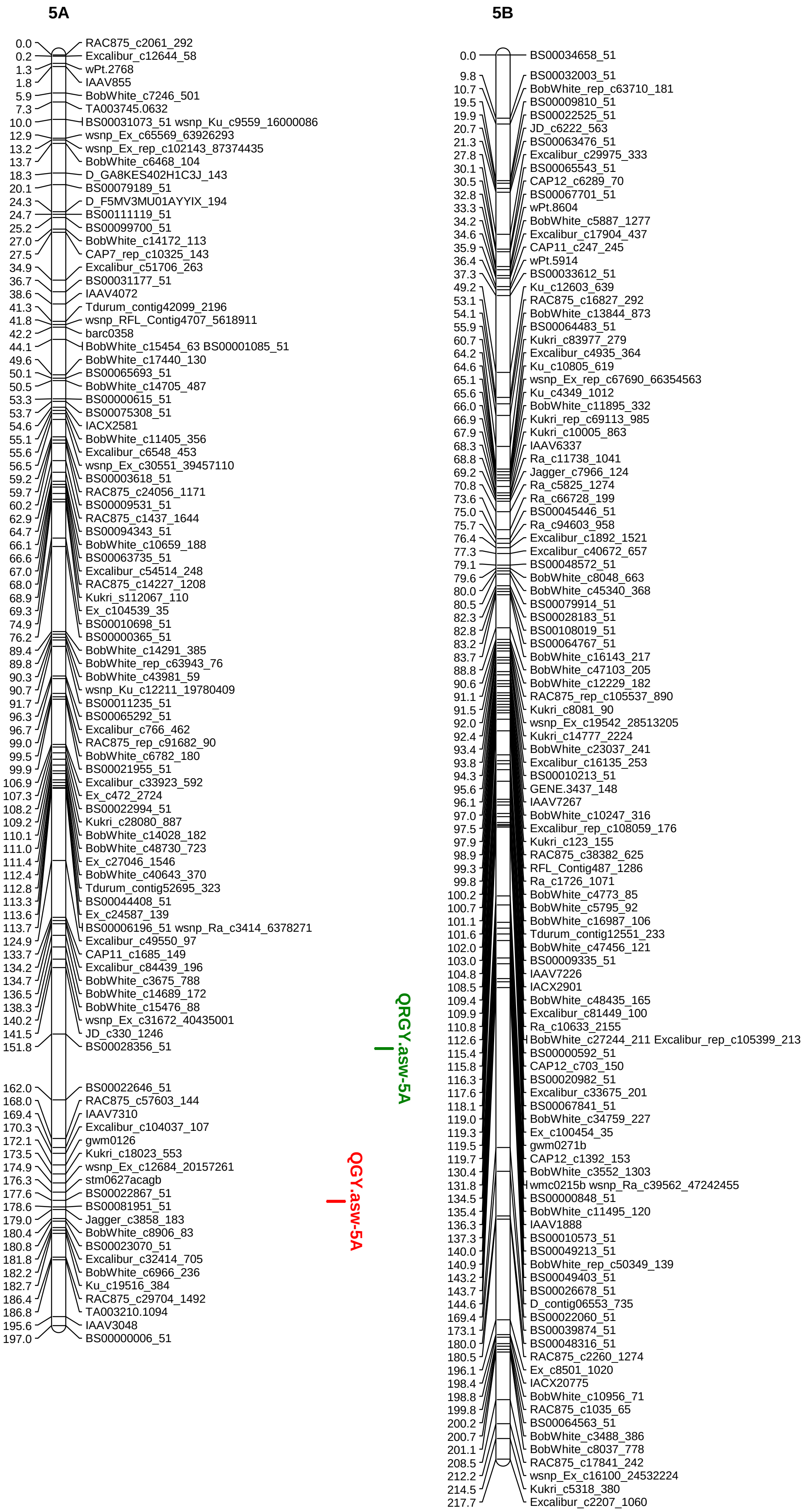
5A
5B
QRGY.asw-5A
QGY.asw-5A
RAC875_c2061_292
Excalibur_c12644_58
wPt.2768
IAAV855
BobWhite_c7246_501
TA003745.0632
BS00031073_51 wsnp_Ku_c9559_16000086
wsnp_Ex_c65569_63926293
wsnp_Ex_rep_c102143_87374435
BobWhite_c6468_104
D_GA8KES402H1C3J_143
BS00079189_51
D_F5MV3MU01AYYIX_194
BS00111119_51
BS00099700_51
BobWhite_c14172_113
CAP7_rep_c10325_143
Excalibur_c51706_263
BS00031177_51
IAAV4072
Tdurum_contig42099_2196
wsnp_RFL_Contig4707_5618911
barc0358
BobWhite_c15454_63 BS00001085_51
BobWhite_c17440_130
BS00065693_51
BobWhite_c14705_487
BS00000615_51
BS00075308_51
IACX2581
BobWhite_c11405_356
Excalibur_c6548_453
wsnp_Ex_c30551_39457110
BS00003618_51
RAC875_c24056_1171
BS00009531_51
RAC875_c1437_1644
BS00094343_51
BobWhite_c10659_188
BS00063735_51
Excalibur_c54514_248
RAC875_c14227_1208
Kukri_s112067_110
Ex_c104539_35
BS00010698_51
BS00000365_51
BobWhite_c14291_385
BobWhite_rep_c63943_76
BobWhite_c43981_59
wsnp_Ku_c12211_19780409
BS00011235_51
BS00065292_51
Excalibur_c766_462
RAC875_rep_c91682_90
BobWhite_c6782_180
BS00021955_51
Excalibur_c33923_592
Ex_c472_2724
BS00022994_51
Kukri_c28080_887
BobWhite_c14028_182
BobWhite_c48730_723
Ex_c27046_1546
BobWhite_c40643_370
Tdurum_contig52695_323
BS00044408_51
Ex_c24587_139
BS00006196_51 wsnp_Ra_c3414_6378271
Excalibur_c49550_97
CAP11_c1685_149
Excalibur_c84439_196
BobWhite_c3675_788
BobWhite_c14689_172
BobWhite_c15476_88
wsnp_Ex_c31672_40435001
JD_c330_1246
BS00028356_51
BS00022646_51
RAC875_c57603_144
IAAV7310
Excalibur_c104037_107
gwm0126
Kukri_c18023_553
wsnp_Ex_c12684_20157261
stm0627acagb
BS00022867_51
BS00081951_51
Jagger_c3858_183
BobWhite_c8906_83
BS00023070_51
Excalibur_c32414_705
BobWhite_c6966_236
Ku_c19516_384
RAC875_c29704_1492
TA003210.1094
IAAV3048
BS00000006_51
BS00034658_51
BS00032003_51
BobWhite_rep_c63710_181
BS00009810_51
BS00022525_51
JD_c6222_563
BS00063476_51
Excalibur_c29975_333
BS00065543_51
CAP12_c6289_70
BS00067701_51
wPt.8604
BobWhite_c5887_1277
Excalibur_c17904_437
CAP11_c247_245
wPt.5914
BS00033612_51
Ku_c12603_639
RAC875_c16827_292
BobWhite_c13844_873
BS00064483_51
Kukri_c83977_279
Excalibur_c4935_364
Ku_c10805_619
wsnp_Ex_rep_c67690_66354563
Ku_c4349_1012
BobWhite_c11895_332
Kukri_rep_c69113_985
Kukri_c10005_863
IAAV6337
Ra_c11738_1041
Jagger_c7966_124
Ra_c5825_1274
Ra_c66728_199
BS00045446_51
Ra_c94603_958
Excalibur_c1892_1521
Excalibur_c40672_657
BS00048572_51
BobWhite_c8048_663
BobWhite_c45340_368
BS00079914_51
BS00028183_51
BS00108019_51
BS00064767_51
BobWhite_c16143_217
BobWhite_c47103_205
BobWhite_c12229_182
RAC875_rep_c105537_890
Kukri_c8081_90
wsnp_Ex_c19542_28513205
Kukri_c14777_2224
BobWhite_c23037_241
Excalibur_c16135_253
BS00010213_51
GENE.3437_148
IAAV7267
BobWhite_c10247_316
Excalibur_rep_c108059_176
Kukri_c123_155
RAC875_c38382_625
RFL_Contig487_1286
Ra_c1726_1071
BobWhite_c4773_85
BobWhite_c5795_92
BobWhite_c16987_106
Tdurum_contig12551_233
BobWhite_c47456_121
BS00009335_51
IAAV7226
IACX2901
BobWhite_c48435_165
Excalibur_c81449_100
Ra_c10633_2155
BobWhite_c27244_211 Excalibur_rep_c105399_213
BS00000592_51
CAP12_c703_150
BS00020982_51
Excalibur_c33675_201
BS00067841_51
BobWhite_c34759_227
Ex_c100454_35
gwm0271b
CAP12_c1392_153
BobWhite_c3552_1303
wmc0215b wsnp_Ra_c39562_47242455
BS00000848_51
BobWhite_c11495_120
IAAV1888
BS00010573_51
BS00049213_51
BobWhite_rep_c50349_139
BS00049403_51
BS00026678_51
D_contig06553_735
BS00022060_51
BS00039874_51
BS00048316_51
RAC875_c2260_1274
Ex_c8501_1020
IACX20775
BobWhite_c10956_71
RAC875_c1035_65
BS00064563_51
BobWhite_c3488_386
BobWhite_c8037_778
RAC875_c17841_242
wsnp_Ex_c16100_24532224
Kukri_c5318_380
Excalibur_c2207_1060

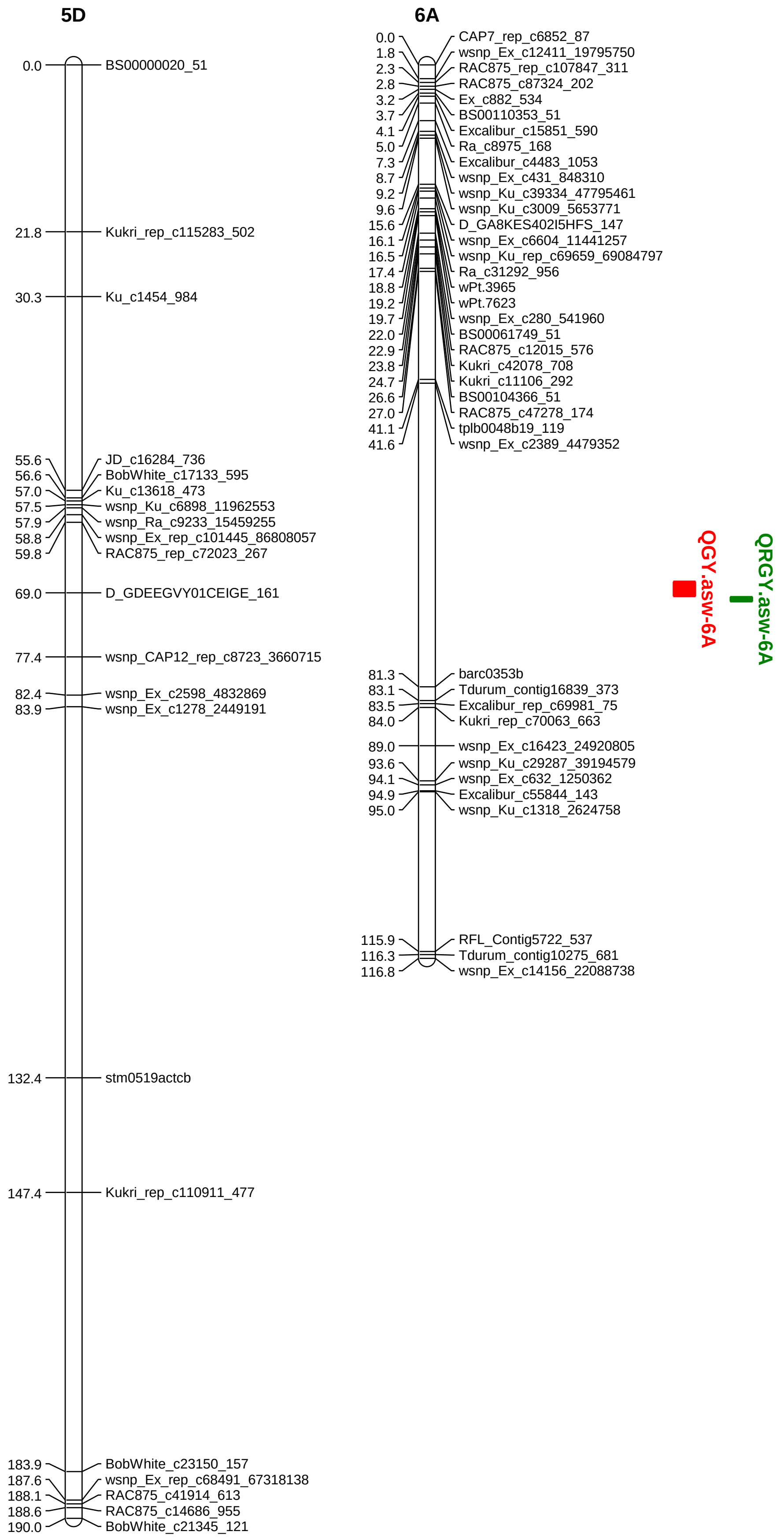
5D
0.0 BS00000020_51
21.8 Kukri_rep_c115283_502
30.3 Ku_c1454_984
55.6 JD_c16284_736
56.6 BobWhite_c17133_595
57.0 Ku_c13618_473
57.5 wsnp_Ku_c6898_11962553
57.9 wsnp_Ra_c9233_15459255
58.8 wsnp_Ex_rep_c101445_86808057
59.8 RAC875_rep_c72023_267
69.0 D_GDEEGVY01CEIGE_161
77.4 wsnp_CAP12_rep_c8723_3660715
82.4 wsnp_Ex_c2598_4832869
83.9 wsnp_Ex_c1278_2449191
132.4 stm0519actcb
147.4 Kukri_rep_c110911_477
183.9 BobWhite_c23150_157
187.6 wsnp_Ex_rep_c68491_67318138
188.1 RAC875_c41914_613
188.6 RAC875_c14686_955
190.0 BobWhite_c21345_121
6A
0.0 CAP7_rep_c6852_87
1.8 wsnp_Ex_c12411_19795750
2.3 RAC875_rep_c107847_311
2.8 RAC875_c87324_202
3.2 Ex_c882_534
3.7 BS00110353_51
4.1 Excalibur_c15851_590
5.0 Ra_c8975_168
7.3 Excalibur_c4483_1053
8.7 wsnp_Ex_c431_848310
9.2 wsnp_Ku_c39334_47795461
9.6 wsnp_Ku_c3009_5653771
15.6 D_GA8KES402I5HFS_147
16.1 wsnp_Ex_c6604_11441257
16.5 wsnp_Ku_rep_c69659_69084797
17.4 Ra_c31292_956
18.8 wPt.3965
19.2 wPt.7623
19.7 wsnp_Ex_c280_541960
22.0 BS00061749_51
22.9 RAC875_c12015_576
23.8 Kukri_c42078_708
24.7 Kukri_c11106_292
26.6 BS00104366_51
27.0 RAC875_c47278_174
41.1 tplb0048b19_119
41.6 wsnp_Ex_c2389_4479352
81.3 barc0353b
83.1 Tdurum_contig16839_373
83.5 Excalibur_rep_c69981_75
84.0 Kukri_rep_c70063_663
89.0 wsnp_Ex_c16423_24920805
93.6 wsnp_Ku_c29287_39194579
94.1 wsnp_Ex_c632_1250362
94.9 Excalibur_c55844_143
95.0 wsnp_Ku_c1318_2624758
115.9 RFL_Contig5722_537
116.3 Tdurum_contig10275_681
116.8 wsnp_Ex_c14156_22088738
QGY.asw-6A
QRGY.asw-6A

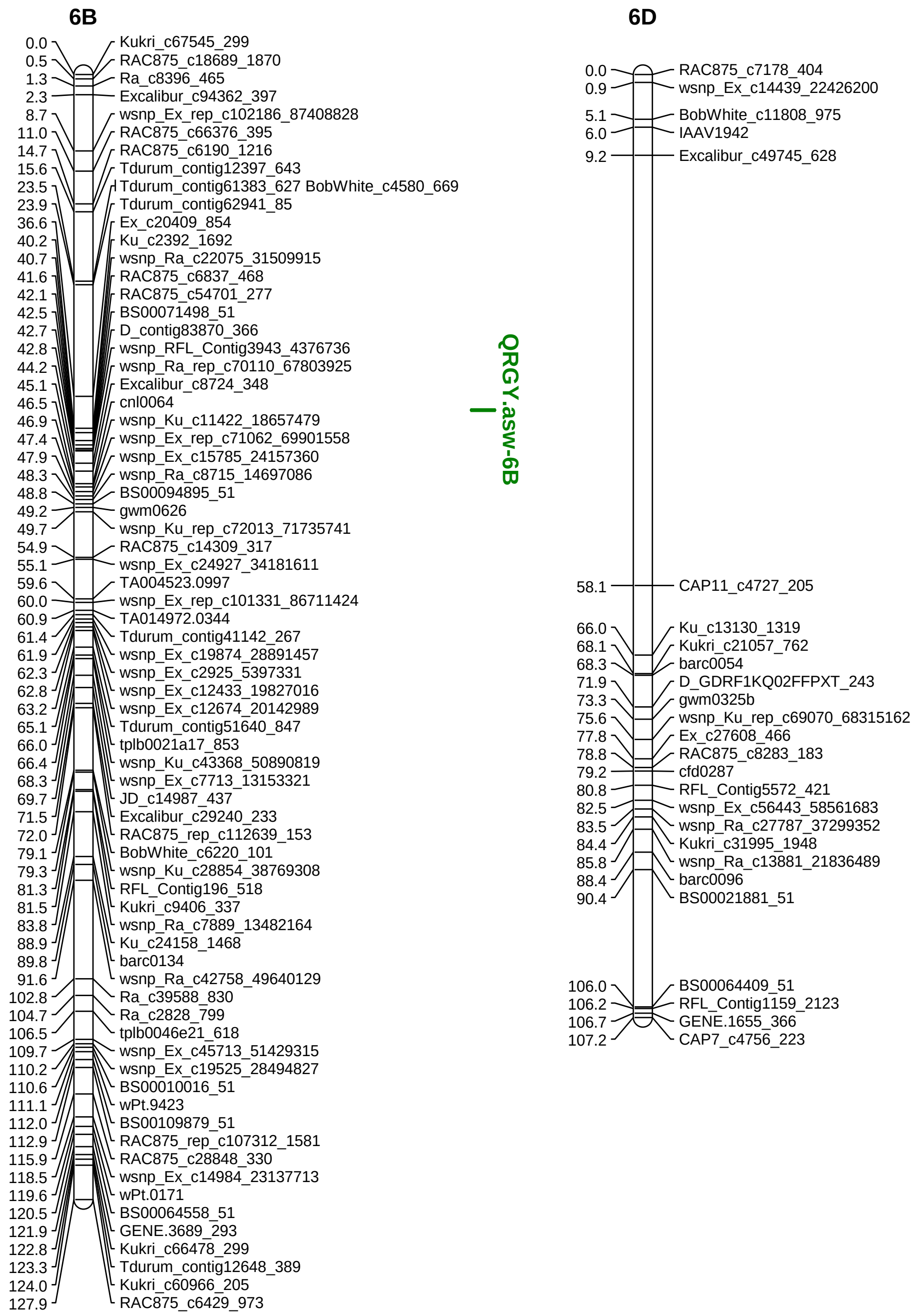
6B
0.0 Kukri_c67545_299
0.5 RAC875_c18689_1870
1.3 Ra_c8396_465
2.3 Excalibur_c94362_397
8.7 wsnp_Ex_rep_c102186_87408828
11.0 RAC875_c66376_395
14.7 RAC875_c6190_1216
15.6 Tdurum_contig12397_643
23.5 Tdurum_contig61383_627 BobWhite_c4580_669
23.9 Tdurum_contig62941_85
36.6 Ex_c20409_854
40.2 Ku_c2392_1692
40.7 wsnp_Ra_c22075_31509915
41.6 RAC875_c6837_468
42.1 RAC875_c54701_277
42.5 BS00071498_51
42.7 D_contig83870_366
42.8 wsnp_RFL_Contig3943_4376736
44.2 wsnp_Ra_rep_c70110_67803925
45.1 Excalibur_c8724_348
46.5 cnl0064
46.9 wsnp_Ku_c11422_18657479
47.4 wsnp_Ex_rep_c71062_69901558
47.9 wsnp_Ex_c15785_24157360
48.3 wsnp_Ra_c8715_14697086
48.8 BS00094895_51
49.2 gwm0626
49.7 wsnp_Ku_rep_c72013_71735741
54.9 RAC875_c14309_317
55.1 wsnp_Ex_c24927_34181611
59.6 TA004523.0997
60.0 wsnp_Ex_rep_c101331_86711424
60.9 TA014972.0344
61.4 Tdurum_contig41142_267
61.9 wsnp_Ex_c19874_28891457
62.3 wsnp_Ex_c2925_5397331
62.8 wsnp_Ex_c12433_19827016
63.2 wsnp_Ex_c12674_20142989
65.1 Tdurum_contig51640_847
66.0 tplb0021a17_853
66.4 wsnp_Ku_c43368_50890819
68.3 wsnp_Ex_c7713_13153321
69.7 JD_c14987_437
71.5 Excalibur_c29240_233
72.0 RAC875_rep_c112639_153
79.1 BobWhite_c6220_101
79.3 wsnp_Ku_c28854_38769308
81.3 RFL_Contig196_518
81.5 Kukri_c9406_337
83.8 wsnp_Ra_c7889_13482164
88.9 Ku_c24158_1468
89.8 barc0134
91.6 wsnp_Ra_c42758_49640129
102.8 Ra_c39588_830
104.7 Ra_c2828_799
106.5 tplb0046e21_618
109.7 wsnp_Ex_c45713_51429315
110.2 wsnp_Ex_c19525_28494827
110.6 BS00010016_51
111.1 wPt.9423
112.0 BS00109879_51
112.9 RAC875_rep_c107312_1581
115.9 RAC875_c28848_330
118.5 wsnp_Ex_c14984_23137713
119.6 wPt.0171
120.5 BS00064558_51
121.9 GENE.3689_293
122.8 Kukri_c66478_299
123.3 Tdurum_contig12648_389
124.0 Kukri_c60966_205
127.9 RAC875_c6429_973
QRGY.asw-6B
6D
0.0 RAC875_c7178_404
0.9 wsnp_Ex_c14439_22426200
5.1 BobWhite_c11808_975
6.0 IAAV1942
9.2 Excalibur_c49745_628
58.1 CAP11_c4727_205
66.0 Ku_c13130_1319
68.1 Kukri_c21057_762
68.3 barc0054
71.9 D_GDRF1KQ02FFPXT_243
73.3 gwm0325b
75.6 wsnp_Ku_rep_c69070_68315162
77.8 Ex_c27608_466
78.8 RAC875_c8283_183
79.2 cfd0287
80.8 RFL_Contig5572_421
82.5 wsnp_Ex_c56443_58561683
83.5 wsnp_Ra_c27787_37299352
84.4 Kukri_c31995_1948
85.8 wsnp_Ra_c13881_21836489
88.4 barc0096
90.4 BS00021881_51
106.0 BS00064409_51
106.2 RFL_Contig1159_2123
106.7 GENE.1655_366
107.2 CAP7_c4756_223

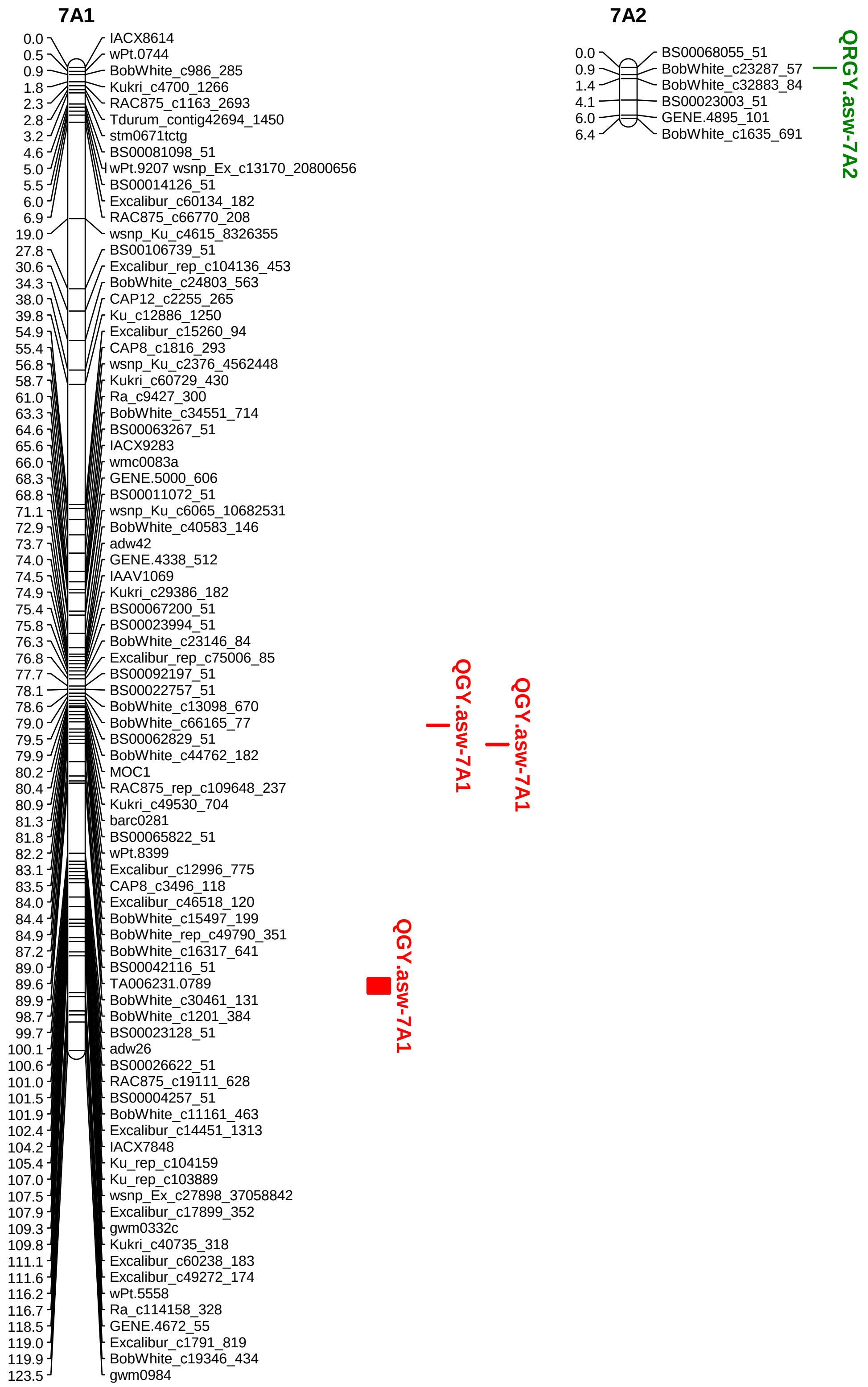
7A1
0.0 IACX8614
0.5 wPt.0744
0.9 BobWhite_c986_285
1.8 Kukri_c4700_1266
2.3 RAC875_c1163_2693
2.8 Tdurum_contig42694_1450
3.2 stm0671tctg
4.6 BS00081098_51
5.0 wPt.9207 wsnp_Ex_c13170_20800656
5.5 BS00014126_51
6.0 Excalibur_c60134_182
6.9 RAC875_c66770_208
19.0 wsnp_Ku_c4615_8326355
27.8 BS00106739_51
30.6 Excalibur_rep_c104136_453
34.3 BobWhite_c24803_563
38.0 CAP12_c2255_265
39.8 Ku_c12886_1250
54.9 Excalibur_c15260_94
55.4 CAP8_c1816_293
56.8 wsnp_Ku_c2376_4562448
58.7 Kukri_c60729_430
61.0 Ra_c9427_300
63.3 BobWhite_c34551_714
64.6 BS00063267_51
65.6 IACX9283
66.0 wmc0083a
68.3 GENE.5000_606
68.8 BS00011072_51
71.1 wsnp_Ku_c6065_10682531
72.9 BobWhite_c40583_146
73.7 adw42
74.0 GENE.4338_512
74.5 IAAV1069
74.9 Kukri_c29386_182
75.4 BS00067200_51
75.8 BS00023994_51
76.3 BobWhite_c23146_84
76.8 Excalibur_rep_c75006_85
77.7 BS00092197_51
78.1 BS00022757_51
78.6 BobWhite_c13098_670
79.0 BobWhite_c66165_77
79.5 BS00062829_51
79.9 BobWhite_c44762_182
80.2 MOC1
80.4 RAC875_rep_c109648_237
80.9 Kukri_c49530_704
81.3 barc0281
81.8 BS00065822_51
82.2 wPt.8399
83.1 Excalibur_c12996_775
83.5 CAP8_c3496_118
84.0 Excalibur_c46518_120
84.4 BobWhite_c15497_199
84.9 BobWhite_rep_c49790_351
87.2 BobWhite_c16317_641
89.0 BS00042116_51
89.6 TA006231.0789
89.9 BobWhite_c30461_131
98.7 BobWhite_c1201_384
99.7 BS00023128_51
100.1 adw26
100.6 BS00026622_51
101.0 RAC875_c19111_628
101.5 BS00004257_51
101.9 BobWhite_c11161_463
102.4 Excalibur_c14451_1313
104.2 IACX7848
105.4 Ku_rep_c104159
107.0 Ku_rep_c103889
107.5 wsnp_Ex_c27898_37058842
107.9 Excalibur_c17899_352
109.3 gwm0332c
109.8 Kukri_c40735_318
111.1 Excalibur_c60238_183
111.6 Excalibur_c49272_174
116.2 wPt.5558
116.7 Ra_c114158_328
118.5 GENE.4672_55
119.0 Excalibur_c1791_819
119.9 BobWhite_c19346_434
123.5 gwm0984
QGY.asw-7A1
QGY.asw-7A1
QGY.asw-7A1
7A2
0.0 BS00068055_51
0.9 BobWhite_c23287_57
1.4 BobWhite_c32883_84
4.1 BS00023003_51
6.0 GENE.4895_101
6.4 BobWhite_c1635_691
QRGY.asw-7A2

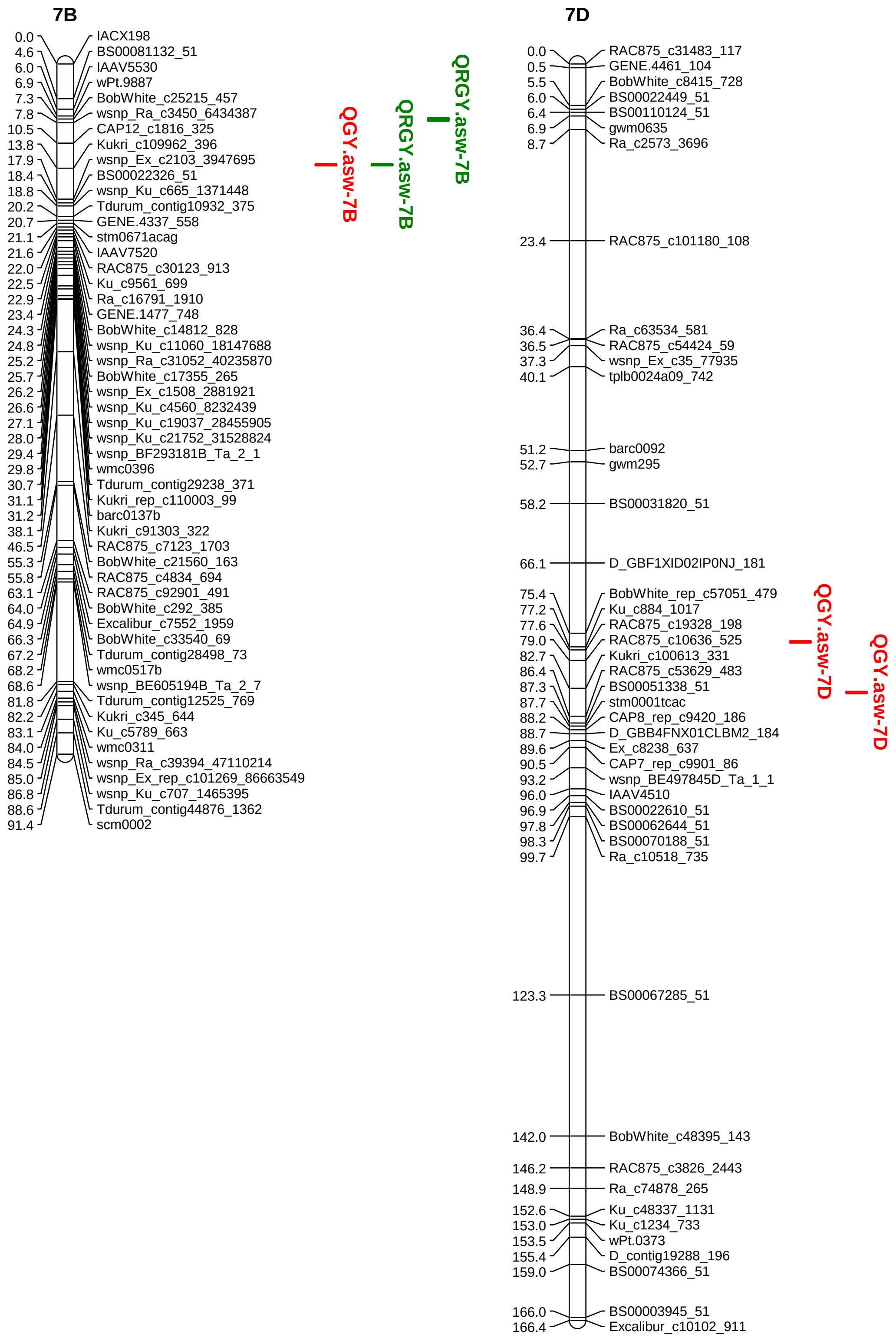

7B
7D
QGY.asw-7B
QRGY.asw-7B
QRGY.asw-7B
QGY.asw-7D
QGY.asw-7D

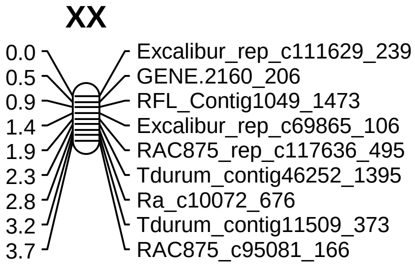
XX
0.0 Excalibur_rep_c111629_239
0.5 GENE.2160_206
0.9 RFL_Contig1049_1473
1.4 Excalibur_rep_c69865_106
1.9 RAC875_rep_c117636_495
2.3 Tdurum_contig46252_1395
2.8 Ra_c10072_676
3.2 Tdurum_contig11509_373
3.7 RAC875_c95081_166
