# Supplementary material for: Genetic Basis for Variation in Wheat Grain Yield in Response to Varying Nitrogen Application
Source: PLoS One. 2016 Jul 26;11(7):e0159374. doi: 10.1371/journal.pone.0159374 (PMC4961366; doi:10.1371/journal.pone.0159374)
Supplement: S1 Table — (DOCX) [file pone.0159374.s002.docx]

**Supporting Information**

**Genetic Basis for Variation in Wheat Grain Yield in Response to Varying Nitrogen Application**

Saba Mahjourimajd^1^, Julian Taylor ^3^, Beata Sznajder^1^, Andy Timmins^1^, Fahimeh Shahinnia^1, #a^, Zed Rengel^4^, Hossein Khabaz-Saberi^4^, Haydn Kuchel^2^, Mamoru Okamoto^1*^, Peter Langridge^1*^

^1^Australian Centre for Plant Functional Genomics (ACPFG), The University of Adelaide, PMB1, Glen Osmond, SA 5064, Australia

^2^Australian Grain Technologies, PMB1, Glen Osmond, SA 5064, Australia

^3^School of Agriculture, Food and Wine, Waite Research Institute, The University of Adelaide, PMB 1, Glen Osmond, SA 5064, Australia

^4^Soil Science and Plant Nutrition M087, School of Earth and Environment, University of Western Australia, 35 Stirling Highway, Crawley WA 6009, Australia

*Present address:*

^#a^Current Address: Leibniz-Institute of Plant Genetics and Crop Plant Research (IPK), Corrensstr. 3, 06466 Gatersleben, Germany

**S1 Table. Heritability analysis of the sites for grain yield (GY, kg ha^-1^) at varying nitrogen (N) treatments**

| **Site** | **N treatment**  **(kg ha^-1^)** | **Heritability** |
| --- | --- | --- |
| LAM 12 | 18 | 0.00 |
| LAM 12 | 52 | 0.18 |
| LAM 12 | 87 | 0.26 |
| PIN 11 | 0 | 0.75 |
| PIN 11 | 75 | 0.90 |
| PIN 11 | 150 | 0.87 |
| PIN 12 | 0 | 0.56 |
| PIN 12 | 75 | 0.71 |
| PIN 12 | 150 | 0.56 |
| YAN 11 | 0 | 0.50 |
| YAN 11 | 75 | 0.33 |
| YAN 11 | 150 | 0.39 |
| ED 13 | 0 | 0.71 |
| ED 13 | 60 | 0.78 |
| WH 13 | 0 | 0.73 |
| WH 13 | 35 | 0.78 |
