# Supplementary material for: Genetic Basis for Variation in Wheat Grain Yield in Response to Varying Nitrogen Application
Source: PLoS One. 2016 Jul 26;11(7):e0159374. doi: 10.1371/journal.pone.0159374 (PMC4961366; doi:10.1371/journal.pone.0159374)
Supplement: S2 Table — (DOCX) [file pone.0159374.s003.docx]

**Supporting Information**

**Genetic Basis for Variation in Wheat Grain Yield in Response to Varying Nitrogen Application**

Saba Mahjourimajd^1^, Julian Taylor ^3^, Beata Sznajder^1^, Andy Timmins^1^, Fahimeh Shahinnia^1, #a^, Zed Rengel^4^, Hossein Khabaz-Saberi^4^, Haydn Kuchel^2^, Mamoru Okamoto^1*^, Peter Langridge^1*^

^1^Australian Centre for Plant Functional Genomics (ACPFG), The University of Adelaide, PMB1, Glen Osmond, SA 5064, Australia

^2^Australian Grain Technologies, PMB1, Glen Osmond, SA 5064, Australia

^3^School of Agriculture, Food and Wine, Waite Research Institute, The University of Adelaide, PMB 1, Glen Osmond, SA 5064, Australia

^4^Soil Science and Plant Nutrition M087, School of Earth and Environment, University of Western Australia, 35 Stirling Highway, Crawley WA 6009, Australia

*Present address:*

^#a^Current Address: Leibniz-Institute of Plant Genetics and Crop Plant Research (IPK), Corrensstr. 3, 06466 Gatersleben, Germany

**S2 Table. Genomic regions underlying the single effect of nitrogen (N) on heading date (HD), relative anthesis (RA) and relative maturity (RM), adjoining markers, peak position (cM), logarithm of odds (LOD), *R^2^* (%) and additive effect in various Australian sites**

| **Chr.** | **Trait** | **N treatment** | **Site and year** | **Adjoining markers** | **Position**  **(cM)** | **LOD** | ***R^2^***  **(%)** | **Allele effect** |
| --- | --- | --- | --- | --- | --- | --- | --- | --- |
| 2A | RM | N87 | LAM 12 | *BobWhite_c1049_338 −* ***wsnp_Ex_rep_c69799_68760822*** | 87.2 | 4.4 | 10 | 0.86 |
| 2B | RA | N75 | PIN 12 | ***Tdurum_contig54634_956*** *− TA001874.1495* | 2.3 | 5.3 | 8 | 0.92 |
|  | RM | N52 | LAM 12 | *wsnp_JD_c23434_20022750 −* ***RAC875_c22997_534*** | 13.7 | 4.5 | 9 | 0.74 |
| 2B | HD | N150 | YAN 11 | *CAP12_c3807_144 −* ***Kukri_c26288_419*** | 21.7 | 8.7 | 12 | -1.48 |
|  | HD | N75 | YAN 11 | *CAP12_c3807_144 −* ***Kukri_c26288_419*** | 22.7 | 6.8 | 9 | -1.28 |
|  | HD | N0 | YAN 11 | *CAP12_c3807_144 −* ***Kukri_c26288_419*** | 23.4 | 7.5 | 11 | -1.48 |
|  | RM | N18 | LAM 12 | *CAP12_c3807_144 −* ***Kukri_c26288_419*** | 23.4 | 4 | 8 | 0.57 |
| 2D | RM | N52 | LAM 12 | *tplb0057n10_689 −* ***RAC875_c24201_984*** | 35.5 | 10.2 | 23 | -1.27 |
|  | HD | N0 | YAN 11 | *tplb0057n10_689 −* ***RAC875_c24201_984*** | 36.1 | 16.1 | 28 | 2.41 |
|  | RA | N0 | PIN 12 | *tplb0057n10_689 −* ***RAC875_c24201_984*** | 36.1 | 5.8 | 13.2 | -1.08 |
|  | RA | N75 | PIN 12 | *tplb0057n10_689 −* ***RAC875_c24201_984*** | 36.1 | 18.2 | 36 | -2.02 |
|  | RM | N87 | LAM 12 | *tplb0057n10_689 −* ***RAC875_c24201_984*** | 36.1 | 5.6 | 12 | -1.09 |
|  | HD | N75 | YAN 11 | *tplb0057n10_689 −* ***RAC875_c24201_984*** | 37.1 | 26.5 | 52 | 3.06 |
|  | HD | N150 | YAN 11 | *tplb0057n10_689 −* ***RAC875_c24201_984*** | 37.1 | 28.2 | 52 | 3.19 |
|  | RA | N150 | PIN 12 | *tplb0057n10_689 −* ***RAC875_c24201_984*** | 37.1 | 18.5 | 37 | -2.14 |
|  | RM | N18 | LAM 12 | *tplb0057n10_689 −* ***RAC875_c24201_984*** | 38.1 | 9.2 | 21 | -0.96 |
| 5B | RM | N52 | LAM 12 | *RAC875_c2260_1274 −* ***Ex_c8501_1020*** | 195.7 | 4 | 8 | -0.64 |
| 6A | RM | N18 | LAM 12 | ***wsnp_Ex_c2389_4479352*** *− barc0353b* | 59.5 | 3.7 | 10 | -0.56 |
| 7A1 | RA | N0 | PIN 12 | *Ku_c12886_1250 −* ***Excalibur_c15260_94*** | 47.5 | 3.7 | 9.8 | 0.84 |
|  | HD | N150 | YAN 11 | *Ku_c12886_1250 −* ***Excalibur_c15260_94*** | 52.4 | 5.3 | 7 | -1.01 |
|  | HD | N75 | YAN 11 | *Ku_c12886_1250 −* ***Excalibur_c15260_94*** | 52.8 | 7 | 9 | -1.12 |
|  | HD | N0 | YAN 11 | *BS00011072_51 −* ***wsnp_Ku_c6065_10682531*** | 71 | 3.7 | 5 | -0.89 |
| 7B | HD | N75 | YAN 11 | ***IACX198*** *− BS00081132_51* | 0 | 3.4 | 4 | -0.74 |
|  | HD | N0 | YAN 11 | ***IACX198*** *− BS00081132_51* | 2 | 6.5 | 10 | -1.25 |
